# Personalized‐Context‐Aware Age Gap: A New Multi‐Omics Measurement Based on Age‐Enhanced Model AOE‐Net for Aging Acceleration and Chronic Disease Risk Prediction

**DOI:** 10.1111/acel.70552

**Published:** 2026-05-28

**Authors:** Feng‐Ao Wang, Tao Zeng, Chunchun Yuan, Hongyu Wang, Yule Yu, Enjin Deng, Yao Wang, Jiangxun Ji, Jiarui Cui, Dezhi Tang, Ruikun He, Yongjun Wang, Yixue Li

**Affiliations:** ^1^ Bioland Laboratory Guangzhou China; ^2^ Key Laboratory of Systems Health Science of Zhejiang Province, School of Life Science, Hangzhou Institute for Advanced Study University of Chinese Academy of Sciences Hangzhou China; ^3^ Guangzhou National Laboratory Guangzhou China; ^4^ GMU‐GIBH Joint School of Life Sciences, Guangdong Provincial Key Laboratory of Protein Modification and Disease, The Guangdong‐Hong Kong‐Macao Joint Laboratory for Cell Fate Regulation and Diseases Guangzhou Medical University Guangzhou China; ^5^ Longhua Hospital Shanghai University of Traditional Chinese Medicine Shanghai China; ^6^ Key Laboratory of Theory and Therapy of Muscles and Bones Ministry of Education Shanghai China; ^7^ College of Animal Science and Technology Guangxi University Nanning China; ^8^ BYHEALTH Institute of Nutrition & Health Guangzhou China; ^9^ Shanghai University of Traditional Chinese Medicine Shanghai China

**Keywords:** age gap, chronic disease, contrastive learning, multi‐omics, pre‐training model

## Abstract

Aging is a global issue that affects human health and increases disease risk. The traditional concept of the “age gap (AG),” defined as the difference between estimated biological age and an individual's chronological age, has been used for self‐monitoring the risk of age‐related diseases. However, the current AG does not account for the stratified aging patterns across different stages of chronological age, which may lead to biased or paradoxical interpretations of aging acceleration. To address these limitations, we propose Personalized‐context‐Aware Age Gap (PAAG), a robust metric to estimate aging acceleration, based on our new pre‐training model AOE‐Net (Age Order Enhanced Network). AOE‐Net employs age‐order enhanced contrastive learning on multi‐omics data from healthy populations to learn latent representations that accurately reconstruct aging trajectories by capturing biological deviation rather than technical deviation in omics data. We demonstrate that PAAG, generated via fine‐tuning AOE‐Net, significantly outperforms AG of conventional first‐ and second‐generation aging clocks in predicting clinical outcomes. This superior predictive power was validated across diverse age‐related diseases and phenotypes: pan‐cancer (overall survival), subclinical atherosclerosis (PESA score), and osteoporosis (bone mineral density). Crucially, PAAG serves as a context‐aware metric that may improve the clinical outcome prediction of existing aging clocks. Furthermore, interpretive analysis of PAAG's molecular drivers revealed a strong functional enrichment for immune‐response pathways, providing a shared mechanistic link between accelerated aging and disease. Collectively, PAAG could serve as a stable indicator of aging acceleration for clinically assessing age‐related diseases, and AOE‐Net provides an effective pre‐training model for aging study and PAAG evaluation.

## Graphical abstract

Addressing systemic biases in traditional aging clocks, we introduce PAAG, a context‐aware multi‐omics metric derived from the pre‐trained AOE‐Net. PAAG accurately captures localized aging acceleration, demonstrating superior predictive power for chronic disease risks and clinical outcomes across diverse aging populations.

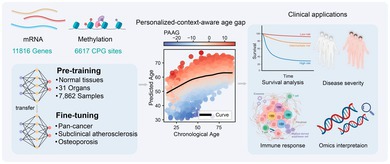

## 1 Introduction

Aging is a complex process characterized by a gradual decline in bodily functions and the accumulation of damage at the cellular and molecular levels (Lopez‐Otin et al. 2013; Rutledge et al. 2022). This decline affects cells, tissues, organs, and ultimately the entire body, influenced by a combination of genetic, epigenetic, and environmental factors (Harman 1981; Jin et al. 2024). As a growing global health concern, aging is placing substantial strain on healthcare systems worldwide because it significantly increases the risk of chronic diseases such as heart disease, neurodegenerative disorders, and various types of cancer (Kabacik et al. 2022; Lopez‐Otin et al. 2023; Sniderman and Furberg 2008; Wang et al. 2025). Understanding an individual's aging process is crucial for assessing overall health (National Research Council 2001). Traditionally, aging has been measured by chronological age—the number of years a person has lived since birth (Kotter‐Grühn et al. 2015). However, this method does not account for the varying rates at which individuals age biologically, since some may age faster or slower than their chronological age suggests (Sanderson and Scherbov 2010). Thus, there is a need for more accurate measures of biological age, which can better reflect an individual's real dynamical aging process rather than simply the stable and continuous passage of living time (Ahadi et al. 2020; Fleischer et al. 2018; Jylhävä et al. 2017; Zhang et al. 2019).

Recent advances in the high‐throughput sequencing technologies have provided a large number of multi‐omics data (Liu et al. 2023; Wei et al. 2024; Yuan et al. 2024; Zhang et al. 2022) such as DNA methylation and gene expression, enabling biological age estimation at the molecular level. The first generation of aging clocks, exemplified by the pan‐tissue DNA methylation clocks developed by Horvath (2013) and Hannum et al. (2013), were trained to predict chronological age with remarkable accuracy, which are known as chronological‐age‐trained models. In these omics aging clocks, the concept of the “age gap”—the difference between estimated biological age and chronological age—has become increasingly important in aging research. Especially, this AG measurement usually serves as an indicator of aging acceleration (Jawinski et al. 2022; Zhu et al. 2023), where a positive AG indicates the biological age exceeds chronological age and suggests accelerated aging and increased risks of disease and mortality. However, these first‐generation clocks faced a conceptual paradox of aging estimation: as their accuracy in predicting chronological age increased, the biological meaning of biological age and their “age gap” diminished.

To overcome these limitations, the second‐generation clocks, such as PhenoAge (Levine et al. 2018) and GrimAge (Lu et al. 2019), were introduced, utilizing clinical and mortality outcomes to train DNA methylation models. These models strengthened the link between aging‐related predictions and functional decline, but they provided agent of aging clock and did not fully resolve how age‐related deviation should be interpreted across different chronological‐age contexts. In parallel, aging‐clock development has expanded to diverse molecular modalities (e.g., transcriptome or methylation) and modeling strategies (e.g., specific tissue or organ), including transcriptomic clocks such as BiT age clock (Meyer and Schumacher 2021), inflammatory and deep‐learning‐based models such as iAge (Sayed et al. 2021), proteins‐centric frameworks (Proietti et al. 2023) and DNA‐methylation‐based models such as DeepMAge (Galkin et al. 2021). These studies illustrate the breadth of the field, but they also highlight the importance of distinguishing among models with different inputs, prediction targets, and biological scopes.

Recent aging‐clock research has expanded from single‐modality models to multi‐omics frameworks for constructing aging clocks, reflecting the view that aging is a system‐level process that may not be fully captured by any single molecular layer. These studies seek to integrate complementary information across DNA methylation, transcriptomics, proteomics, metabolomics, microbiome features, and related clinical or phenotypic measurements. For multi‐omics based aging clocks, some frameworks combine molecular and clinical domains to better characterize healthspan and disease risk. OMICmAge (Chen et al. 2026), for instance, uses epigenetic biomarker proxies to represent proteomic, metabolomic, and clinical domains while remaining quantifiable from DNA methylation data alone. Other studies (Nie et al. 2022) have used broader multimodal profiling to estimate aging across organs or physiological systems, incorporating clinical tests, immune repertoire, targeted metabolomics, gut microbiome, physical fitness, and skin‐related features. Population‐based integrative studies (Chen et al. 2024; Li, Xiong, et al. 2023) have further combined transcripts, proteins, metabolites, microbes, and clinical laboratory measurements to derive both global and customized aging clocks, thereby emphasizing the heterogeneity of aging across individuals and biological systems.

Crucially, the application of multi‐omics to longitudinal cohorts has advanced our understanding of the dynamic pace of aging. For instance, deep longitudinal profiling (Ahadi et al. 2020) quantified individual molecular aging rates to define distinct “ageotypes” (e.g., hepatic, immune, or metabolic), revealing that specific biological systems age at different speeds within the same individual. Furthermore, recent longitudinal multi‐omics analyses (Shen et al. 2024) have demonstrated that human aging is not an entirely gradual process; instead, it exhibits non‐linear dynamics with substantial molecular “bursts”—periods of accelerated molecular dysregulation occurring at specific life stages, particularly during the mid‐40s and early 60s. Overall, these developments suggest that multi‐omics integration can provide a richer description of aging‐related biology, while also introducing challenges related to high dimensionality, incomplete modality availability, and model generalizability.

Despite these advances, important challenges remain in both the modeling of multi‐omics data for aging‐clock construction and the interpretation of age‐related deviation. In first‐generation clocks trained on chronological age, age gap is typically defined as the difference between predicted biological age and chronological age. In second‐generation clocks, similar deviation measures can also be calculated relative to chronological age; however, because these models are trained on phenotypic or mortality‐related targets, the biological meaning of such deviations is not fully equivalent to that of chronological‐age‐trained clocks. More importantly, the conventional age‐gap framework remains limited by two related essential issues. First, in chronological‐age‐trained models, it is susceptible to regression‐to‐the‐mean bias, which tends to overestimate the age of younger individuals and underestimate that of older individuals. Second, it assumes that the same deviation measure has the same biological meaning across different stages of life, thereby ignoring life‐stage‐specific context and potentially obscuring clinically relevant differences in age‐related deviation. Beyond these conceptual issues, multi‐omics aging‐clock construction also faces practical challenges. Multi‐omics data are highly heterogeneous across modalities, are frequently incomplete in real‐world cohorts, and typically contain far more features than samples (Hasin et al. 2017), all of which complicate data integration, increase over‐fitting risk, and reduce model generalizability (Wang et al. 2024; Withnell et al. 2021). In addition, although deep learning models can capture complex nonlinear age‐related patterns, many remain difficult to interpret, thereby limiting biological insight and the discovery of aging‐associated molecular markers. These limitations together motivate the development of approaches that can integrate incomplete high‐dimensional multi‐omics data while also improving the interpretation of age‐related deviation.

With the above motivations, recent advances in foundation models which utilize large‐scale pre‐training demonstrate greater robustness and adaptability (Cui et al. 2024; Pai et al. 2024; Xu et al. 2024) and can offer a promising solution to address the above conceptual and computational challenges (Ma et al. 2024). Inspired by the representation learning strategies, we propose a new personalized‐context‐aware age gap (PAAG) metric instead of conventional AG to enhance multi‐omics aging clock and develop a deep learning framework AOE‐Net (Age‐Order Enhanced Network) to learn biological age for PAAG estimation. On one hand, PAAG is a local, group‐relative measure of age‐related deviation, whereas the conventional age gap functions as a more global measure. Thus, PAAG can provide a new approach to measure individual aging acceleration within the same age group, which calculates the average predicted age for each chronological age group as an “anchor” and determines an individual's adjusted age gap by comparing their predicted ages to this anchor rather than original chronological age itself. On the other hand, AOE‐Net can support PAAG estimation, which first learns a shared low‐dimensional latent representation from high‐dimensional multi‐omics data and then introduces chronological‐age‐bin structure as age stratification guidance into this latent space during model pre‐training. Specifically, age‐order‐enhanced contrastive learning guides samples from the same chronological‐age‐bin to be closer in latent space while preserving ordinal relationships across chronological‐age‐bins. After this pre‐training step, the model is ready and can be fine‐tuned for biological‐age prediction, and PAAG is then computed as the deviation of an individual's predicted biological age from the mean predicted biological age within the corresponding chronological‐age group. Thus, PAAG itself is independent of model optimization during pre‐training, and AOE‐Net learns an age‐structured representation that supports more stable and interpretable downstream PAAG estimation.

Based on comprehensive case studies on multi‐omics datasets related to different aging‐related chronic diseases (including cancer, subclinical atherosclerosis, and osteoporosis), AOE‐Net and PAAG demonstrated higher precision in estimating aging acceleration and significant correlations with diverse clinical phenotypes. PAAG serves as a broadly applicable metric that can strengthen the clinical application of existing first‐ and second‐generation aging clocks. This highlights the core potential of PAAG and AOE‐Net together as a new “AI for aging” tool for precise clinical health assessment, paving the way for personalized aging management and targeted interventions against complex diseases.

## 2 Results

### 2.1 Framework of AOE‐Net and PAAG

**FIGURE 1:**
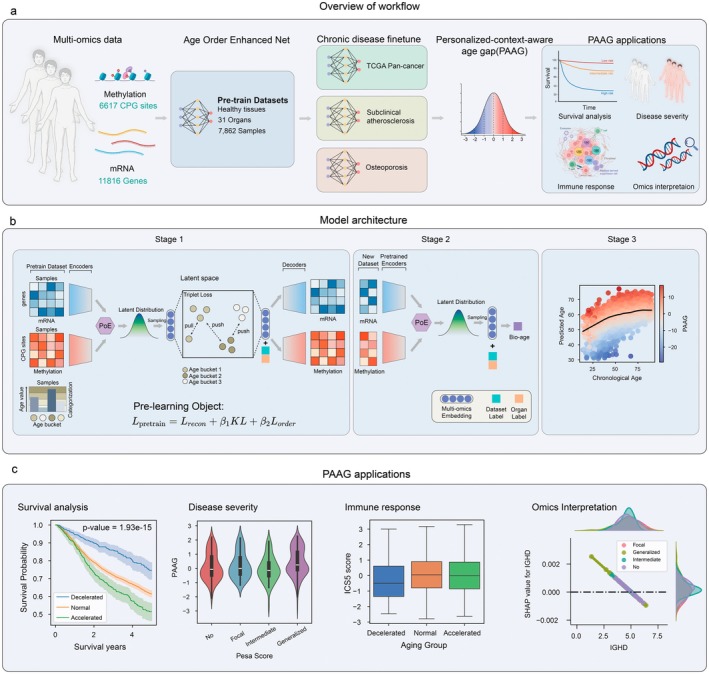
Design of AOE‐Net and PAAG for aging acceleration and chronic disease risk prediction. (a) Diagram of the whole workflow, including pre‐training and fine‐tuning of AOE‐Net, and the computation of PAAG. PAAG quantifies the age gap relative to a population‐based reference which is different to conventional AG. (b) Detailed architecture of AOE‐Net and the computational pipeline for PAAG. Of note, the Product of Experts (PoE) approach is used to derive the joint latent representation from multi‐omics inputs, thus AOE‐Net can effectively leverage both unpaired and paired data during pre‐training and fine‐tuning stages. Especially in this work, AOE‐Net is pre‐trained on normal samples, encompassing unpaired RNA‐seq and DNA methylation profiles from the GTEx dataset, alongside paired RNA‐seq and DNA methylation data from TCGA normal samples, guided by personalized‐context‐aware enhanced contrastive learning. The model then can be fine‐tuned to predict biological age and compute PAAG. (c) Downstream applications of PAAG in diverse biological and clinical contexts. PAAG is utilized to predict or correlate with different phenotypes, including survival outcome, disease severity, immune response and omics‐based biomarker relevant to both aging and disease.

To accurately measure biological age and estimate aging acceleration based on multi‐omics data, AOE‐Net is first pre‐trained on healthy controls to establish a reference model, and subsequently fine‐tuned on target datasets (e.g., disease cohorts) using transfer learning (Figure 1a,b). PAAG metric is calculated to assess aging acceleration, which can be applied to investigate different aging‐related chronic diseases and investigate the potential associations of PAAG with diverse clinical phenotypes, such as cancer prognosis and immune response (Figure 1c).

AOE‐Net follows a biologically guided three‐phase framework (Figure 1b). In the first phase, the model integrates RNA‐seq and DNA methylation data into a shared low‐dimensional representation, allowing complementary aging‐related information from different biological layers to be captured within a unified framework (see Methods for details). This design also allows the model to remain applicable when only one modality (e.g., only RNA‐seq) is available in a downstream task and dataset. To introduce age‐related structure during pre‐training, samples are grouped into 10‐year chronological‐age bins, and these age groups are used to organize the shared sample representation according to age order in low‐dimensional data space (i.e., latent space). As a result, the learned sample representation captures both multi‐omics information and age‐related structure. In the second phase, AOE‐Net is fine‐tuned to predict biological age from the learned sample representation. In the third phase, PAAG is calculated as a context‐aware alternative to the conventional AG. Specifically, for each chronological‐age bin and its individuals, an anchor age is calculated as the mean predicted biological age of samples within that group, and each individual's PAAG is computed as the deviation of predicted biological age from this group‐specific reference (i.e., mean predicted biological age, Figure 1b). Then, PAAG is used for diverse downstream tasks and analyses, including survival stratification and disease‐severity assessment (Figure 1c). Unlike the conventional age gap which compares an individual's biological age directly with chronological age, PAAG accounts for variation within age groups and therefore provides a more context‐aware measure of age‐related deviation (Figure S1).

### 2.2 Age‐Informed Latent Trajectories Captured by AOE‐Net

AOE‐Net is pre‐trained on normal samples, encompassing RNA‐seq from 7862 tissue samples spanning 31 distinct tissues in the GTEx (RNA‐seq) dataset, alongside paired RNA‐seq and DNA methylation data from TCGA normal samples. By incorporating both multi‐ and uni‐omics data, AOE‐Net acquires a robust multi‐omics representation. As shown in UMAP (Uniform Manifold Approximation and Projection), there is a clear gradual progression of latent embeddings (i.e., low‐dimensional representations) that corresponded to chronological‐age groups (divided into 10‐year intervals based on chronological‐age‐bin structure; see Figure S2a). Actually, the pseudotime trajectories derived from these latent embeddings (Figure S2b) were also strongly associated with chronological age. This supports that the contrastive learning of AOE‐Net can reasonably organize samples by biological age in latent space. Such age‐informed latent space in normal groups provides a solid basis of age‐structure, which allows downstream transfer learning in other individuals/populations (e.g., disease sample groups) by accurate detection of deviations from normal aging patterns caused by disease (i.e., measured by PAAG).

### 2.3 Overall Survival and Immune Response Stratification in Pan‐Cancer Individuals Associated to PAAG Compared to Baseline Methods

**FIGURE 2:**
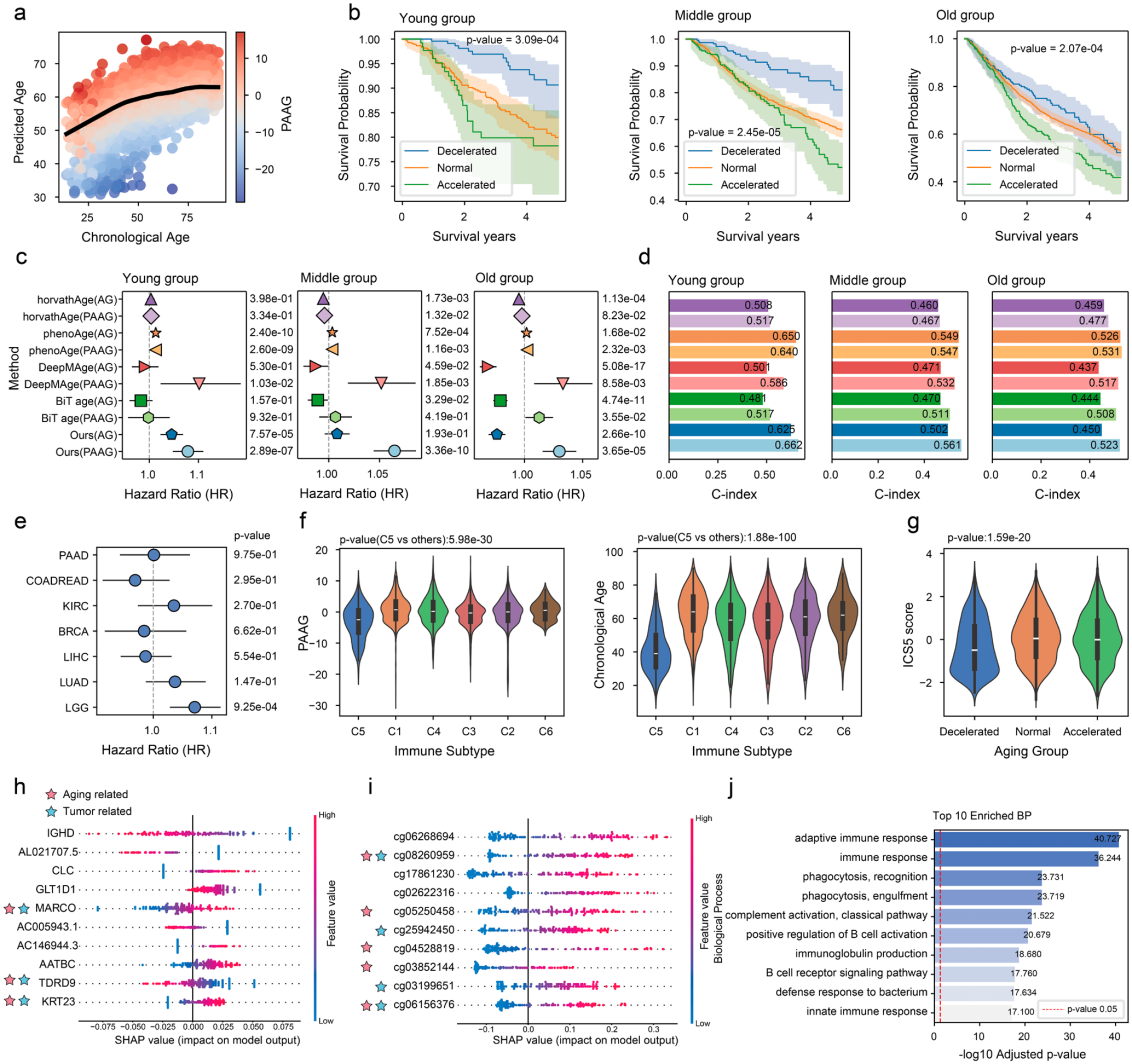
Identifying overall survival and immune response stratification in pan‐cancer populations by AOE‐Net and PAAG. (a) Diagram illustrating the relationship between chronological age and predicted biological age by AOE‐Net, with PAAG quantifying the deviation for population reference. (b) Kaplan–Meier survival curves stratified by PAAG‐defined aging groups (decelerated, normal, and accelerated) across pan‐cancer patients, analyzed within young (< 40 years), middle‐aged (40–60 years), and old (> 60 years) chronological age subgroups. The aging‐accelerated individuals consistently show the poorest overall survival. *p*‐values for each subgroup are shown (log‐rank test). (c) Hazard Ratios (HR) for overall survival prediction using various omics aging clocks (e.g., DeepMAge, BiT age clock, PhenoAge and horvathAge) compared to AOE‐Net‐derived PAAG along with ablation experiment. Results are presented for young, middle, and old age subgroups. (d) Concordance Index (C‐index) for overall survival prediction using different omics aging clocks (DeepMAge, BiT age clock, PhenoAge and horvathAge) compared to AOE‐Net‐derived PAAG. Results are presented for young, middle, and old age subgroups. PAAG consistently shows superior predictive accuracy, especially in the young and middle age subgroups. (e) Hazard ratios (HR) comparison for pan‐cancer overall survival analysis using different age regression methods combined with new PAAG or conventional AG, in a manner of ablation study too. (f) The differential distributions of biological age (PAAG) and chronological age across tumor immune subtypes. (g) The variation of ICS5 scores across PAAG‐defined aging groups (Kruskal‐Wallis *p* = 1.59 × 10^−20^). (h) Top 10 genes ranked by SHAP values according to their contribution to PAAG. (i) Top 10 DNA methylation sites ranked by SHAP values according to their contribution to PAAG. (j) The biological processes (BP) significantly enriched in the top 100 SHAP‐prioritized RNA‐seq genes together with their correlated/co‐expressed genes.

To evaluate the clinical relevance of aging acceleration defined by PAAG in cancer, AOE‐Net is first fine‐tuned in 9621 pan‐cancer samples from The Cancer Genome Atlas (TCGA), each of which has paired RNA‐seq and DNA methylation profiles. The model's performance in predicting biological age was evaluated using five‐fold cross‐validation (Figure 2a). To disentangle the effects of biological aging from chronological age, we used PAAG to stratify patients into aging‐decelerated, aging‐normal, and aging‐accelerated groups, performing this analysis independently in three chronological age subgroups: young (< 40 years), middle‐aged (40–60 years), and old (> 60 years). There were significant differences in overall survival among these groups (Kaplan–Meier Multivariate Logrank Test, *p* = 3.09 × 10^−4^ in young group, *p* = 2.45 × 10^−5^ in middle‐aged group and *p* = 2.07 × 10^−4^ in old group; Figure 2b–d), and the aging‐accelerated group consistently had the worst prognosis. This finding, aligning with previous studies (Levine et al. 2018; Li, Chen, et al. 2023; Qiu et al. 2023), demonstrates that PAAG is a potent prognostic biomarker whose utility is independent of a patient's chronological age or age stratification. This prognostic power was evident across diverse cancer types, for example, there was a particularly strong association between accelerated aging and poor outcomes in low‐grade glioma (*p* = 9.25 × 10^−4^, Figure 2e).

To rigorously benchmark the prognostic power of aging acceleration in this well‐known largest cancer dataset, we further compared our AOE‐Net‐derived PAAG against a panel of state‐of‐the‐art first‐ and second‐generation single‐modality aging clocks (e.g., DeepMAge, BiT age clock, PhenoAge, and horvathAge) derived with conventional AG metrics. For such benchmark comparison, we retrained the DeepMAge and BiT age clock models de novo on the respective TCGA methylation and transcriptomic data. Indeed, the original PhenoAge and horvathAge models were primarily developed using blood‐based methylation settings, whose original training contexts differ from the downstream TCGA application presented here. Thus they were calculated from TCGA tumor DNA methylation data using the published methylclock package (Pelegí‐Sisó et al. 2021) similar to recent related studies. Overall, PAAG consistently outperformed all other methods almost across all age cohorts, exhibiting the highest Hazard Ratio (HR) for risk stratification (Figure 2c) and the highest concordance index (C‐index) for survival prediction especially in young and middle‐aged subgroups (Figure 2d), which were also consistent with the multimodal pan‐cancer TCGA study (Chen et al. 2022). To further determine whether this superior performance was attributable to the PAAG framework itself, rather than solely AOE‐Net model, we conducted an ablation study. Remarkably, when we applied our PAAG instead of AG for the other clocks, it substantially enhanced their prognostic power, surpassing the performance of their original AG metrics (Figure 2c,d). This key result demonstrates that the PAAG metric is not specific to AOE‐Net and can be applied to predictions from other clocks and represents a fundamentally more robust method for quantifying age acceleration relevant to prognosis, which also offers a broadly applicable measurement tool to improve existing aging clocks.

To further demonstrate the versatility and extensibility of the AOE‐Net framework across multiple molecular modalities, we expanded our analysis to a vignette case including proteomic data. We curated a sub‐cohort of 5787 TCGA pan‐cancer samples possessing paired RNA‐seq, DNA methylation, and reverse‐phase protein array (RPPA) data from a previous study (Li et al. 2024). Leveraging the pre‐trained RNA and DNA methylation encoders, we integrated a protein encoder as a parallel branch to establish a tri‐modal (Protein‐RNA‐Methylation) AOE‐Net. As shown in Figure S3, the incorporation of proteomic data yielded a modest yet consistent improvement in the C‐index for prognostic survival prediction across all chronological age strata (young, middle, and old) compared to the bi‐modal (RNA and methylation) model. Furthermore, the PAAG metric derived from this tri‐modal framework effectively stratified patients into aging‐decelerated, aging‐normal, and aging‐accelerated groups, which still exhibited distinctly separable and highly stratified survival trajectories (Figure S4). These findings underscore the robustness of AOE‐Net in accommodating additional modalities and highlight the potential of integrating proteomic data to construct more comprehensive aging clocks.

We next examined the relationship between PAAG and tumor immunity. Because senescent cells are known to affect immune infiltration in the tumor microenvironment (Calcinotto et al. 2019; Reed et al. 2007) and previous studies suggest that senescent cells in the tumor microenvironment can enhance immune responses against tumor cells (Marin et al. 2023; Zhuang et al. 2025). Thus, we examined whether PAAG was associated with immune‐related phenotypes in the pan‐cancer cohort. Analyzing the established TCGA immune subtypes, our findings revealed that, individuals classified as the previously defined immune subtype C5 (i.e., “immunologically quiet”) (Peng 2022) had the lowest PAAG values (Figure 2f), while other subtypes showed higher PAAG values. Additionally, individuals within immune subtype C5 had the youngest chronological age compared to other subtypes. Meanwhile, the aging‐decelerated group had significantly lower immune checkpoint signature 5 (ICS5) scores (Figure 2g; *p* = 1.59 × 10^−20^).

Importantly, these findings should be interpreted cautiously. The observed association does not by itself indicate that accelerated aging corresponds to a uniformly better immune response. Rather, it suggests that higher PAAG is associated with an altered immune‐related microenvironment in the pan‐cancer setting. To further examine whether elevated PAAG is also accompanied by senescence‐related molecular features, we evaluated a six‐gene senescence‐associated transcriptional panel across the PAAG‐defined groups (Figure S5), including cell‐cycle arrest markers *CDKN1A* (Chu et al. 2004) and *CDKN2A* (Agarwal et al. 2012), senescence‐associated secretory phenotype/inflammatory factors *SERPINE1* (Zhang et al. 2024) and *CXCL8* (Coppé et al. 2008), and broader senescence‐associated secretory or stress‐related factors *GDF15* (Guo et al. 2019) and *IGFBP7* (Siraj et al. 2024). All six genes showed significant group‐wise differences (Kruskal–Wallis test; *CDKN1A*, *p* = 9.03 × 10^−19^; *CDKN2A*, *p* = 1.41 × 10^−5^; *SERPINE1*, *p* = 1.52 × 10^−40^; *CXCL8*, *p* = 1.61 × 10^−55^; *GDF15*, *p* = 1.17 × 10^−41^; *IGFBP7*, *p* = 2.87 × 10^−11^). In parallel, a senescence signature score calculated from these retained senescence‐associated genes was also significantly different across PAAG groups and was highest in the aging‐accelerated group (Figure S6; Kruskal‐Wallis *p* = 1.78 × 10^−45^). Together, these results support an association between higher PAAG, altered immune‐related phenotypes, and stronger senescence‐associated transcriptional features. However, they do not establish a direct mechanistic relationship between senescence and the immune checkpoint patterns observed here. Further work in more refined settings, such as longitudinal or single‐cell analyses, will be needed to clarify these relationships.

Building on this functional association, biomarkers with dual relevance to tumorigenesis and aging were further identified by SHAP (SHapley Additive exPlanations) (Antwarg et al. 2021). On one hand, we selected the top 10 most influential RNA expression features identified by AOE‐Net (Figure 2h, Figure S7), which revealed tumor‐associated genes such as *MARCO* (macrophage receptor regulating tumor microenvironment) (Gu et al. 2023), *KRT23* (epithelial‐mesenchymal transition biomarker for metastasis) (Ren et al. 2020) and *TDRD9* (methylation‐driven oncogenesis) (Guijo et al. 2017). Interestingly, these genes also exhibited associations to aging via distinct mechanisms: *MARCO* in macrophage clearance dysfunction (Son et al. 2023), *TDRD9* in epigenetic regulation of senescence (Rocha‐da‐Silva et al. 2019) and *KRT23* in epithelial aging (Zhang et al. 2017). On the other hand, we also selected the top‐ranked tumor‐associated DNA methylation sites (Figure 2i, Figure S7), which included cg03199651 (*MSX1*) in epithelial‐mesenchymal transition (Chen et al. 2008), cg06156376 (*SHOX2*) in PI3K/AKT pathway activation (Li et al. 2020) and cg25942450 (*TLX3*) in leukemia relevance (Ma et al. 2014), cg04528819 (*KLF14*) in colorectal cancer biomarker (Wu et al. 2019) and cg05250458 (*ZNF177*) in gastric cancer hypermethylation (Mao et al. 2021) identified as potential anti‐cancer markers. Aging‐associated methylation markers comprised cg03852144 (*GLRX*) in oxidative stress (Morgan et al. 2010), cg06156376 (*SHOX2*) in epigenetic clock (Horvath et al. 2020), cg04528819 (*KLF14*) in metabolic aging (Yang and Civelek 2020) and cg05250458 (*ZNF177*) in DNA repair defects (Afzal et al. 2022). These findings demonstrate AOE‐Net's capacity to uncover novel biomarkers with dual potential mechanisms of tumorigenesis and aging. Consistently, Gene Ontology (GO) enrichment analysis based on the top 100 SHAP‐prioritized RNA‐seq genes together with their correlated/co‐expressed genes revealed significant enrichment of immune‐related biological processes (Figure 2j). These results further support the relevance of immune‐associated pathways to PAAG‐related prediction in pan‐cancer.

Besides, we aimed to delineate the conceptual boundary of PAAG, and we evaluated its relationship with longitudinal pace‐of‐aging metrics by comparing PAAG to the DunedinPACE measure (Belsky et al. 2022) within the TCGA pan‐cancer cohorts. As shown in Figure S8, the overall association between the two metrics was modest, displaying only a weak positive trend in the pooled pan‐cancer analysis. Moreover, cancer‐specific analyses revealed substantial heterogeneity across different tumor types (Figure S9). These additional results emphasized that PAAG acts as a distinct, context‐aware measure of cross‐sectional age deviation, and its relationship to longitudinal pace‐of‐aging metrics is inherently limited and highly dependent on the specific disease context.

### 2.4 PAAG in TARGET Pan‐Cancer Cohort Underscores Immune Correlation With Aging in Cancer Patients

**FIGURE 3:**
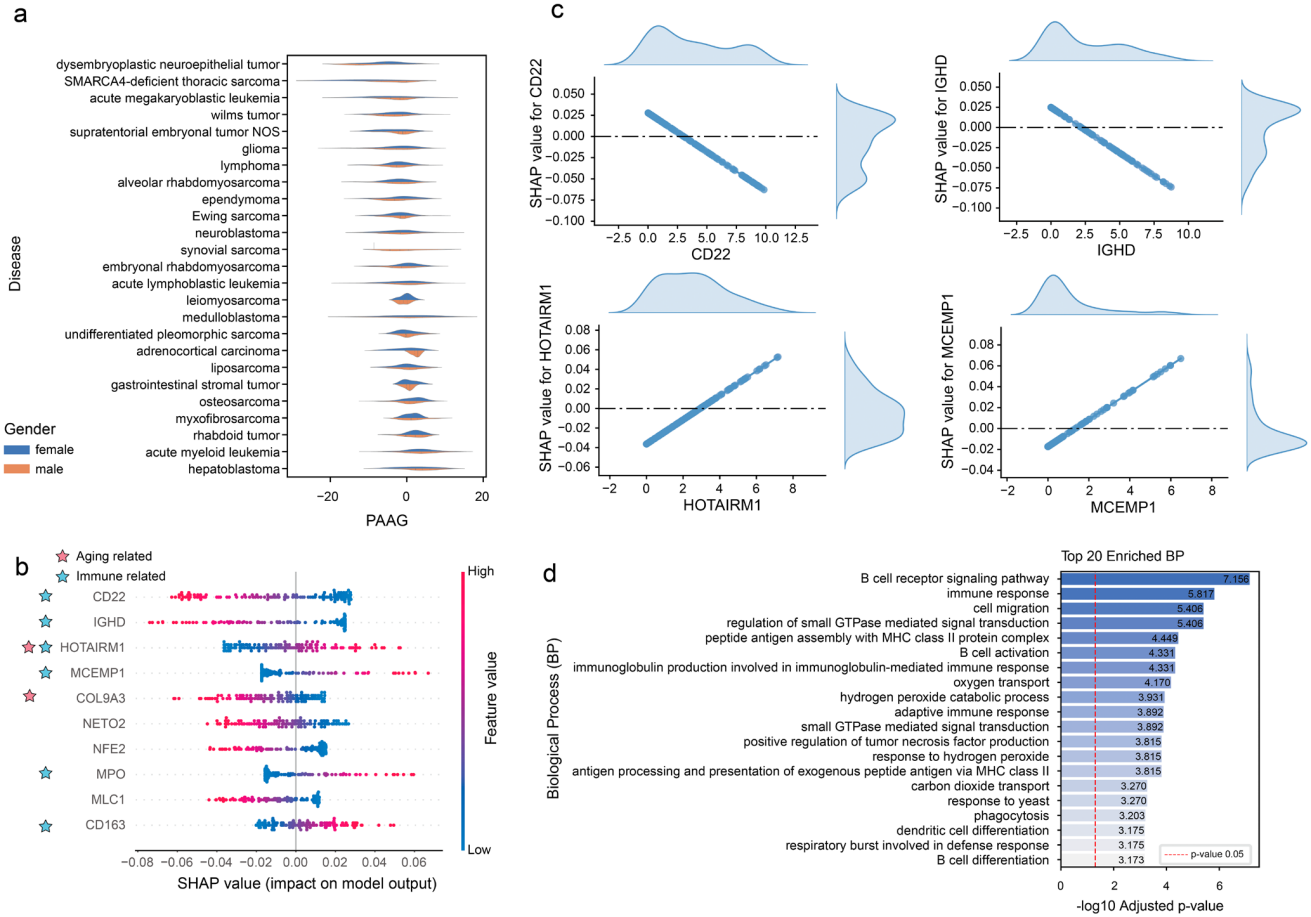
PAAG reveals consistent immune correlation in an independent TARGET pan‐cancer cohort. (a) The distribution of PAAG scores across different cancer types (*n* = 2904 samples), within different gender groups. (b) The top 10 genes ranked by their mean absolute SHAP value, indicating their contribution to the PAAG prediction. (c) SHAP dependence plots for four selected top‐ranking genes (IGHD, HBG2, TCL1A, and IGHG2), showing the relationship between gene expression levels (*x*‐axis) and their corresponding SHAP values (*y*‐axis). (d) Gene Ontology (GO) enrichment analysis for Biological Process (BP) of the top 100 SHAP‐prioritized RNA‐seq genes together with their correlated/co‐expressed genes.

To evaluate the generalizability of AOE‐Net, we applied the above TCGA‐trained model to an independent pan‐cancer dataset (*n* = 2904 samples) from TARGET (Genomics) using only available RNA‐seq data as input. PAAG showed a similar distribution for male and female individuals across different cancer types (Figure 3a). Notably, SHAP analysis identified many immune‐related genes—such as CD22, IGHD, HOTAIRM1, and MCEMP2—as top contributors to the aging prediction (Figure 3b), consistent with our findings in the above TCGA datasets. The identification of *HOTAIRM1* is particularly significant, as literature reports (Ahmadov et al. 2021; Liang et al. 2019) that its high expression is associated with poor prognosis in glioma patients and is explicitly linked to “older age.” Moreover, *HOTAIRM1* overexpression has been found to correlate with “immune activation,” specifically enhanced T‐cell‐mediated immunity and inflammatory responses (Liang et al. 2019). Further supporting these findings, the expression of these top‐ranked genes showed a significant correlation with their corresponding SHAP values (Figure 3c), reaffirming their contribution to aging prediction. Finally, biological process enrichment of the top 100 SHAP‐prioritized RNA‐seq genes together with their correlated/co‐expressed genes uncovered pathways again related to the immune system and immune cell responses (Figure 3d). Together, these results support again a relationship between aging‐related signatures and tumor biology, even in childhood individuals, underscoring the deep connection between immunity and aging within the tumor.

### 2.5 Subclinical Atherosclerosis Severity Characterized by PAAG

In the second case study, the aging‐related cardiovascular risk is explored by examining RNA‐seq data from blood cells of 391 asymptomatic participants in the Progression of Early Subclinical Atherosclerosis (PESA) study (Sanchez‐Cabo et al. 2023). Subclinical atherosclerosis (SA) was identified by the presence of plaques in any of five vascular regions—both carotid arteries, both iliofemoral arteries, or the aorta—evaluated using 2D/3D vascular ultrasound, or by coronary artery calcification (CAC, Agatston score ≥ 0.5) detected through computed tomography (CT) (Aboyans et al. 2010; Fernández‐Ortiz et al. 2013; Ibanez et al. 2021). The extent of SA was classified using the PESA score: no SA, focal SA (one site affected), intermediate SA (two to three sites), and generalized SA (four to six sites).

**FIGURE 4:**
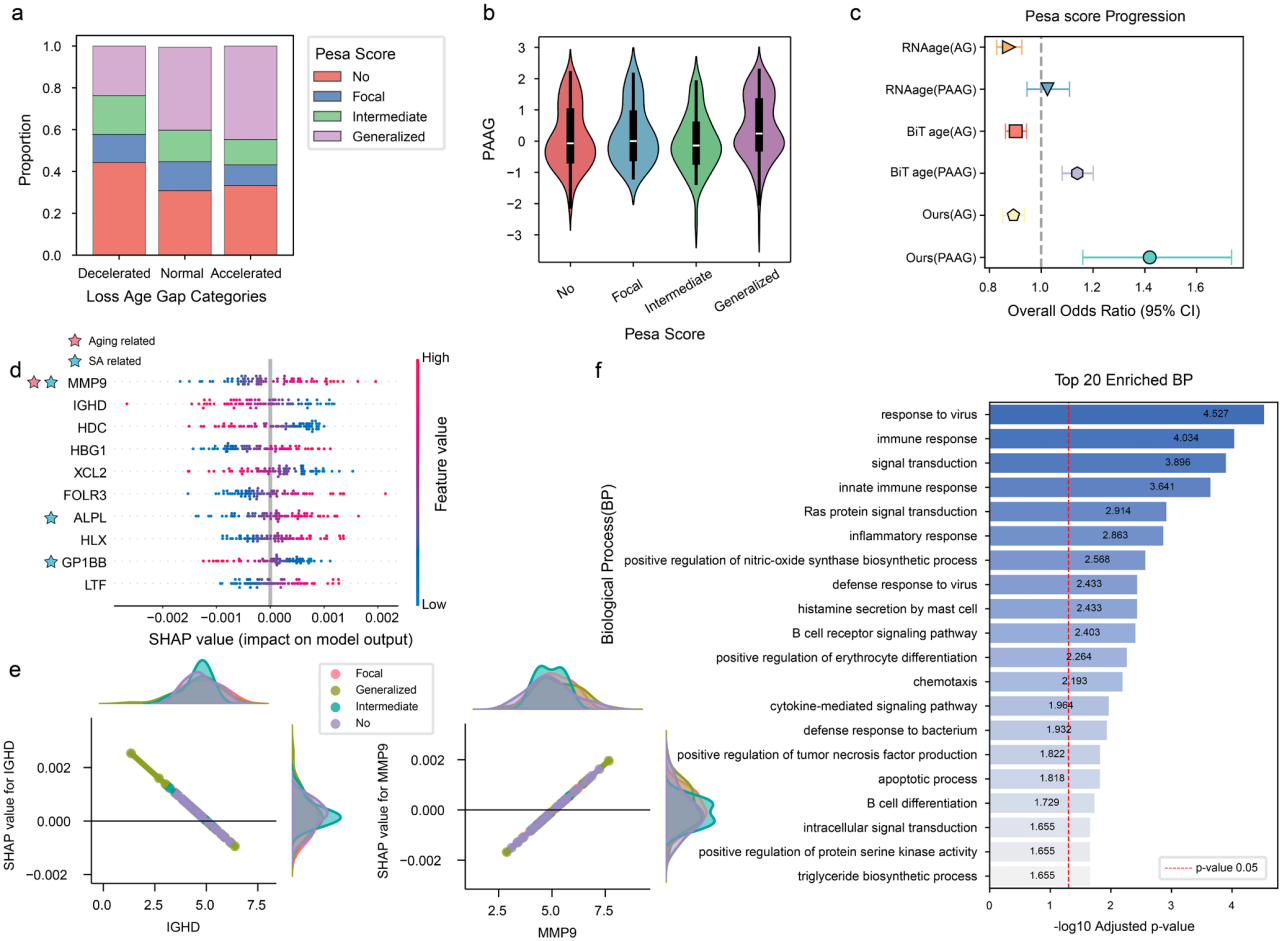
Characterizing subclinical atherosclerosis severity by AOE‐Net and PAAG. (a) Proportion of PESA‐score groups within aging patterns defined by PAAG (decelerated, normal and accelerated aging groups). (b) Distribution of PAAG across four PESA‐score groups categorized by No, Focal, Intermediate and Generalized. (c) Odds ratios for indicating PESA score, evaluated using different methods combined with new PAAG or conventional AG. (d) Top 10 genes ranked by SHAP values for their contribution to PAAG, identifying key molecular contributors. (e) Distribution of SHAP values versus expression levels of *IGHD* or *MMP9*, highlighting their associations with PAAG. (f) The biological processes (BP) significantly associated with the top 100 SHAP‐prioritized RNA‐seq genes together with their correlated/co‐expressed genes, providing new insights into the biological functions linked to PAAG in the context of subclinical atherosclerosis.

After fine‐tuning AOE‐Net with five‐fold cross‐validation, the PAAG for each participant was calculated, according to which asymptomatic participants were then divided into aging‐decelerated, aging‐normal and aging‐accelerated groups. The distributions of PESA scores within each group were then compared and the aging‐accelerated group had a higher proportion of intermediate and generalized SA, indicating an association between aging‐accelerated and increased SA severity (Figure 4a). Likewise, participants with intermediate and generalized SA showed higher PAAG values (Figure 4b). To rigorously benchmark these findings, we compared the predictive power of our AOE‐Net‐derived PAAG framework against established RNA‐based clocks (e.g., BiT age clock and RNAAge), by retraining the BiT age clock model de novo using the PESA transcriptomic data and calculating RNAAge via its published package (Ren and Kuan 2020). Consistently, AOE‐Net‐derived PAAG outperformed the established RNA‐based clocks and achieved the highest odds ratio for identifying individuals with a significant SA burden (Figure 4c). This result establishes PAAG as a promising blood‐based biomarker for subclinical atherosclerosis, highlighting its potential to identify asymptomatic individuals at high risk for future cardiovascular events.

The top 10 genes exhibiting the greatest influence on PAAG, as ranked by SHAP values, were identified (Figure 4d). Changes in their expression levels in peripheral blood cells were found to be associated with generalized SA. Our interpretable analysis highlighted key marker genes implicated in both aging and SA. For instance, *MMP9* encodes a zinc‐dependent endopeptidase that degrades extracellular matrix components, playing a critical role in atherosclerotic plaque destabilization and rupture by weakening the fibrous cap (Bassiouni et al. 2021). *ALPL*, a key regulator of phosphate metabolism, contributes to vascular calcification by promoting hydroxyapatite deposition and the osteogenic transdifferentiation of vascular smooth muscle cells (Liu et al. 2018). *GP1BB*, a subunit of the platelet glycoprotein Ib‐IX‐V complex, facilitates platelet adhesion and thrombus formation by mediating the interaction between platelets and von Willebrand factor at sites of vascular injury (Dib et al. 2022). Notably, *MMP9*, *ALPL*, *and FOLR3* were significantly associated with SA extent at the transcriptional level (*p* ≤ 0.05; Table S1) and were also among the most impactful genes on PAAG (Figure 4d). These findings suggest that these genes may serve as shared biomarkers for both aging and SA (Figure 4e).

Furthermore, we found again that these marker genes are related to the immune system, such as *IGHD* (Pasman et al. 2017), *XCL2* (Chen et al. 2023), *HLX* (Zheng et al. 2004) and *LTF* (Zhao et al. 2021). The top 100 SHAP‐prioritized RNA‐seq genes together with their correlated/co‐expressed genes contributing to PAAG further showed significant functional enrichment in many immune system components (Figure 4f), including immune system processes, response to stimuli and immune responses. Overall, these results suggest common underlying mechanisms linking more severe SA to accelerated aging, that is, innate and adaptive immune pathways, along with the molecules that support their functional associations with aging.

### 2.6 Bone Mineral Density Associated With PAAG for Osteoporosis Individuals

**FIGURE 5:**
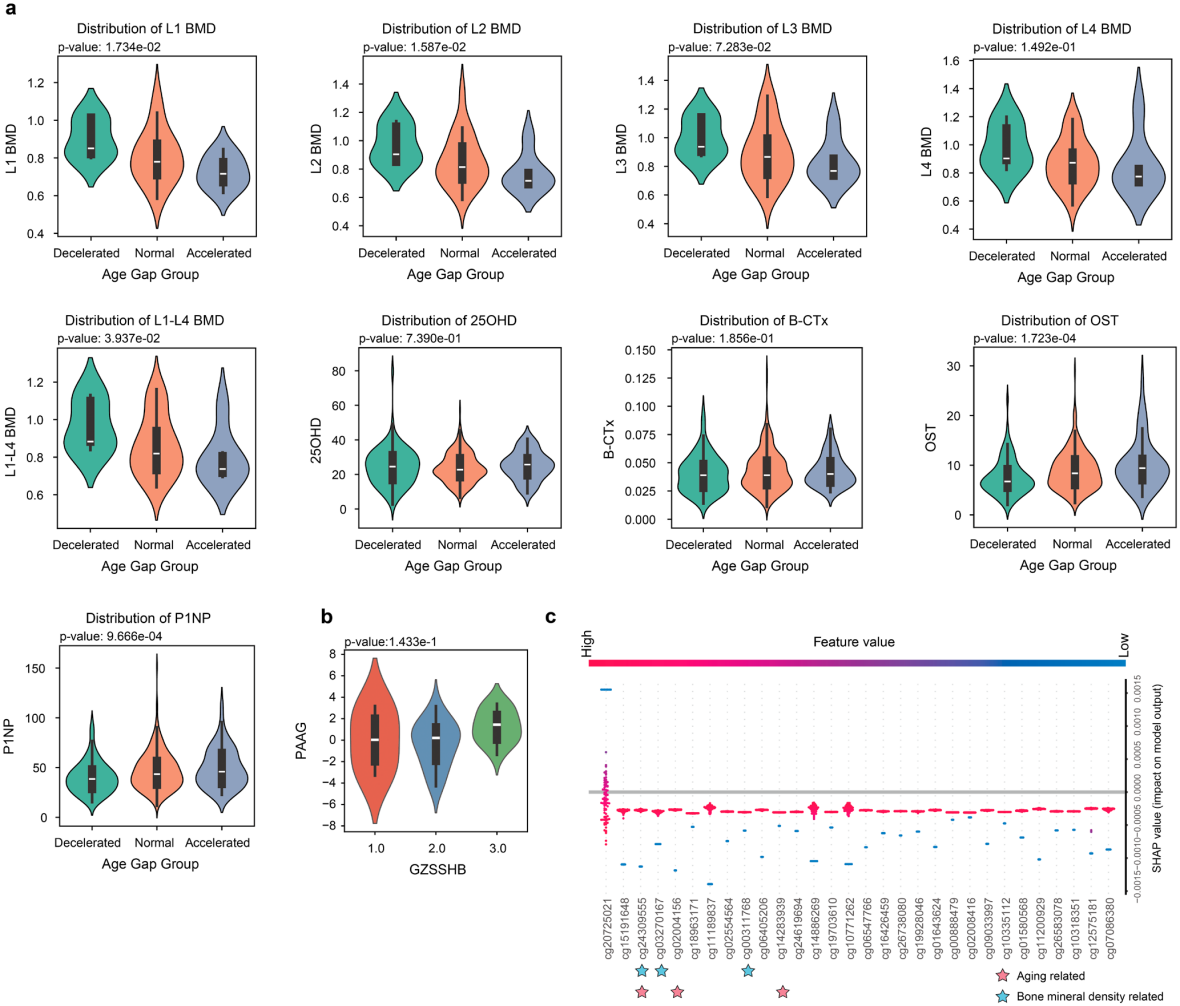
Association between bone mineral density and PAAG for osteoporosis individuals. (a) Distribution of bone mineral density related phenotypes across different aging patterns defined by PAAG (i.e., decelerated, normal and accelerated aging groups). *p*‐values, indicating the significance of the monotonic trend across the ordered groups (decelerated, normal, accelerated), were calculated using the Jonckheere–Terpstra test. (b) Distribution of PAAG among normal, osteopenia and osteoporosis individuals, highlighting disease differences in aging patterns. The *p*‐value indicates the significance of the trend across these ordered clinical groups. (c) Top 30 DNA methylation CpG sites ranked by SHAP values for their contribution to PAAG, identifying key epigenetic markers associated with aging and bone mineral density.

In the third case study, AOE‐Net provided PAAG for a group of 398 older adults including normal, osteopenia, and osteoporosis individuals (Yuan et al. 2024), in which PAAG revealed a systemic relationship between epigenetic aging and bone risk. Accelerated aging was strongly and significantly linked to worsening bone loss, as shown by a negative correlation with lumbar spine bone mineral density (L1–L4 BMD: *p* = 3.937 × 10^−2^) and a positive correlation with serum procollagen type 1 N‐terminal propeptide (P1NP: *p* = 9.666 × 10^−4^). Such biologically accelerated aging would disrupt the balance between bone breakdown and formation (see Figure 5a). When grouped by radiographic severity according to DXA judgments, individuals in the osteoporosis group had higher PAAG values than those within the normal group (Figure 5b).

SHAP value analysis identified the top 30 methylation CpG sites contributing to PAAG (Figure 5c, Figure S10), revealing strong correlations with aging and bone mineral density among cg03270167 (*RAMP1*), cg00311768 (*TSTA3*), cg24309555 (*APOB*), cg02004156 (*TFE3*), cg14283939 (*THSD4*), and cg24619694 (*TAGLN*). *RAMP1* encodes a rate‐limiting protein of the calcitonin receptor‐like receptor (CRLR), which is essential for CRLR activity. In bone metabolism, calcitonin gene‐related peptide (CGRP) regulates osteoblast proliferation and differentiation primarily through CRLR phosphorylation (Zhao et al. 2013). *TSTA3* encodes GDP‐L‐fucose synthase, a key enzyme in GDP‐L‐fucose synthesis during glycosylation, which modulates glycoprotein and proteoglycan modification (Yang et al. 2018). Dysregulation of *TSTA3* may impair osteoblast activity, bone matrix mineralization, and bone mineral density, thereby compromising bone health (Hamamura et al. 2024). *APOB* plays a crucial role in lipid metabolism and cholesterol transport (Panasenko et al. 2005). Additionally, *TFE3* influences cellular homeostasis via the lysosome‐autophagy pathway, and its dysregulation may accelerate metabolic aging (Dang and Back 2021). *THSD4* and *TAGLN* are pivotal in extracellular matrix (ECM) remodeling and vascular function, where ECM degradation may exacerbate tissue aging by contributing to vascular elasticity and structural stability (Mougin et al. 2021). Collectively, *TFE3*, *RAMP1*, and *TSTA3* play central roles in metabolic regulation and hold significant potential as targets for anti‐aging interventions for chronic diseases like osteoporosis.

## 3 Discussion

In this study, we present AOE‐Net, a deep learning framework that uses multi‐omics data, paired with a new PAAG metric for quantifying aging acceleration. They can overcome the limitation that current methods usually underestimate the varying rates at which individuals' age biologically. Across multiple case studies, AOE‐Net and PAAG provide a reliable and interpretable way to measure biological age and aging acceleration, which outperform existing aging clocks in accuracy and clinical relevance across various chronic diseases, including many cancer sites and subclinical atherosclerosis. This pre‐trained personalized‐context‐aware model not only improves how we measure aging but also deepens our insight into the molecular basis of aging and its links to complex diseases.

PAAG improves upon the traditional AG by considering variations within different age groups. By comparing an individual's predicted biological age to the expectation of predicted biological age within their age group, PAAG provides a more accurate measure of aging acceleration. This approach is particularly valuable when studying age‐related diseases, where accelerated aging is often associated with worse clinical outcomes. For instance, in cancer, we observed that the aging‐accelerated group exhibited poorer prognosis, which is consistent with previous research (Li, Chen, et al. 2023; Proietti et al. 2023; Qiu et al. 2023). For another case of subclinical atherosclerosis, accelerated aging as measured by positive PAAG was associated with more severe plaque burden, underscoring again the link between aging acceleration and high cardiovascular risk.

Across several disease settings, we found that the aging‐accelerated group exhibited higher immune response scores, suggesting that accelerated aging is associated with an altered immune microenvironment, particularly in the tumor microenvironment (Hickson et al. 2019; Marin et al. 2023). These findings are consistent with previous studies that senescent cells in the microenvironment may either enhance immune responses or contribute to immune evasion in cancer (Birch and Gil 2020; Pereira et al. 2019; Prata et al. 2018; Reed et al. 2007). Thus, the immune response may serve as a key factor influenced by aging acceleration, and understanding the immune system's role in aging acceleration could pave the way for new therapeutic strategies (Chen and Mellman 2017; Larsen et al. 2022; Wang et al. 2022), which can aim at modulating immune function to mitigate age‐related disease progression. Of note, these current observations suggest a link between accelerated aging and an altered immune microenvironment, which are associative. The mechanistic basis of these associations, particularly with respect to immune efficacy, requires further investigation.

AOE‐Net and PAAG offer a flexible AI framework for studying aging mechanisms, together with SHAP identifying potential biomarkers (e.g., *MMP9*, *IGHD* (Araújo et al. 2018)) for future clinical translation. Clinically, their ability to predict disease risk and severity—shown in cancer, SA and osteoporosis—suggests potential value for future personalized risk stratification, such as targeting immune pathways in patients with accelerated aging. Looking forward, given the longitudinal multi‐omics data, AOE‐Net could further track nonlinear aging changes over time (Shen et al. 2024), and even reveal aging signatures specific to individual cell types when single‐cell multi‐omics data is available (Guo et al. 2021; Hong et al. 2024; Tang et al. 2022; Tang et al. 2024; Zeng and Dai 2019; Zhu et al. 2025). Of course, AOE‐Net trained on larger datasets (e.g., additional protein data) could further boost its reliability, tackling influence caused by high dimensionality and small sample sizes in downstream tasks. Notably, AOE‐Net is robustly designed to handle missing omics data inputs. This feature provides critical practical utility, as acquiring complete multi‐omics profiles in real‐world clinical settings is often unfeasible.

It is important to note that PAAG is inherently a group‐relative metric and does not directly estimate the longitudinal pace of aging within an individual. It is not intended to replace existing pace‐of‐aging metrics, such as DunedinPACE (Belsky et al. 2022) or organ‐specific aging clocks. Indeed, the AOE‐Net‐derived PAAG framework is highly versatile; it can be readily adapted to specific organ or tissue omics data to capture localized aging patterns or potentially support pace‐of‐aging analyses through task‐specific fine‐tuning. Ultimately, its primary utility lies in providing a highly context‐aware interpretation of age‐related deviations within specific chronological peer groups.

Of course, some limitations exist in current study. Relying on multi‐omics data, while effective, is limited by the high cost and complexity of sequencing technologies, which restricts the sample sizes and may introduce selection bias (Jendoubi 2021; López de Maturana et al. 2019). As is well known, the large number of variables from omics data increases the risk of over‐fitting, and the pre‐training of AOE‐Net on diverse cohorts like GTEx by variational autoencoders (VAE) can help reduce this issue (Ballard et al. 2024). Furthermore, the interpretability of VAE can be further enhanced by knowledge distillation (Cho and Hariharan 2019; Gou et al. 2021), incorporating biological and biomedical insights from other foundation models. In addition to anchor age's assumption of similarity within age groups for current PAAG, more complex aging patterns should be suitable measured. This is particularly relevant to recent conceptual advances, such as the finding that accurate aging clocks can be built from accumulating stochastic variation (Meyer and Schumacher 2024). This new paradigm suggests that aging is not merely a programmed shift in mean biomarker levels, but also a progressive loss of regulation leading to increased molecular ‘noise’ or entropy. Therefore, a critical future direction for AOE‐Net is to evolve its VAE architecture to explicitly model this increase in variance and stochasticity of high‐order biological network as a key feature of aging. In addition, future research must expand the scope of evaluation cohorts and longitudinal data modalities. Validation of AOE‐Net in non‐Western or underrepresented populations is still needed to confirm its applicability in complicated clinical settings, because genetic and environmental factors can be both covariates during aging (Pang et al. 2019; Rodríguez‐Rodero et al. 2011). Moreover, incorporating longitudinal tracking and additional omics layers will be vital. As demonstrated by recent studies on longitudinal proteomic clocks—such as the work (Rao et al. 2026)—integrating longitudinal data with complementary modalities like proteomics holds immense future potential to further enhance the predictive accuracy and clinical utility of aging models.

Collectively, AOE‐Net and PAAG will contribute a major advance in attractive aging studies, which can bridge molecular data to clinical outcomes through a personalized‐context‐aware AI approach. PAAG can be further applied for precise health status and disease risk assessment in clinical practice. By moving beyond the constraints of chronological age and traditional aging clocks, this new framework based on pre‐training model provides a powerful “AI for biology” tool for understanding the biology of aging and advancing precision medicine for age‐related diseases.

## 4 Methods

### 4.1 Framework of AOE‐Net

AOE‐Net is a deep generative framework for integrating multi‐omics data and predicting biological age. It comprises two main components: a multi‐omics VAE that learns a shared latent representation across modalities, and an MLP‐based predictor that uses this low‐dimensional representation for downstream estimation and analysis of biological age. The shared latent representation is designed to capture common aging‐related structure from RNA‐seq and DNA methylation data while reducing modality‐specific noise, thereby enabling the model to integrate complementary information across biological layers. The integration of a Product of Experts (PoE) ensures the framework's versatility, enabling it to generalize well to downstream scenarios with missing or single modalities. The sample representation is subsequently fine‐tuned for biological‐age prediction for a particular downstream task (e.g., specific disease context). Importantly, PAAG itself is not optimized directly during pre‐training. Instead, pre‐training encourages the latent representation to reflect age‐related structure, which supports downstream biological‐age prediction and subsequent context‐aware measure estimation of age deviation (i.e., PAAG).

For a single modality (e.g., RNA‐seq), the object of latent deep generative model is to learn the latent space representation and maximize the likelihood of observed data x given latent variables z, defined as:

(1) $$p\left(x;\theta \right)=\int p\left(x|z;\theta \right)p\left(z\right)\mathrm{dz}$$

$p\left(z\right)$ is the prior distribution, typically set as a standard Gaussian distribution, and $p\left(x|z;\theta \right)$ represents the process of generating the observed data from latent variable, and θ denotes the learnable parameters in the decoder. Direct computation of $p\left(x;\theta \right)$ is challenging, a variational posterior $q\left(z|x;\phi \right)$ is introduced to approximate the true posterior distribution $p\left(z|x\right)$, where ϕ are the parameters of data encoder. Using this approximation, Equation (1) can be rewritten as:

(2) $$\text{logp}\left(x;\theta \right)=\log\int p\left(x|z;\theta \right)p\left(z\right)\mathrm{dz}=\log\int q\left(z|x;\phi \right)\frac{p\left(x|z;\theta \right)p\left(z\right)}{q\left(z|x;\phi \right)}\mathrm{dz}$$

Using Jensen's inequality, this leads to the evidence lower bound (ELBO):

(3) $$\text{logp}\left(x;\theta \right)=E_{q\left(z|x;\phi \right)}\left[\log\frac{p\left(z,x\right)}{q\left(z|x;\phi \right)}\right]+\mathrm{KL}\left(q\left(z|x;\phi \right)\|p\left(z|x\right)\right)$$

Since $\mathrm{KL}\left(q\left(z|x;\phi \right)\|p\left(z|x\right)\right)\geq 0$, the ELBO is given by (Doersch 2016):

(4) $$\text{ELBO}=E_{q\left(z|x;\phi \right)}\left[\text{logp}\left(x|z;\theta \right)\right]-\beta *\mathrm{KL}\left(q\left(z|x;\phi \right)\|p\left(z\right)\right)$$

Here, $\mathrm{KL}\left(q\|p\right)$ represents the Kullback–Leibler divergence between $q\left(z|x;\phi \right)$ and prior $p\left(z\right)$.

Then, for the multiple modalities (e.g., multi‐omics data), the modalities are mapped into a shared latent space to estimate the joint latent space distribution. Thus product‐of‐experts (PoE) diagram is incorporated to calculate the joint variational posterior distribution for each omics modality *i* from *N* modalities (i.e., $i\in \left[1,N\right]$) to compute the joint distribution z:

(5) $$q_{\phi }\left(z|X\right)=\prod_{i=1}^{N}q_{\phi_{i}}\left(z|x_{i}\right)$$

The prior distribution of $p\left(z\right)$ is modeled as a standard Gaussian distribution $N\left(z|\mu_{0},\delta_{0}\right)$, and each modality‐specific posterior $q\left(z_{x_{i}}|x_{i}\right)$ is also Gaussian, with mean $\mu_{s_{i}}$ and variance $\delta_{s_{i}}$. The joint distribution z is defined by (Cao and Fleet 2014):

(6) $$\delta ={\left(\delta_{0}^{-1}+\sum_{i\in \left[1,N\right]}\delta_{i}^{-1}\right)}^{-1}$$

(7) $$\mu =\left(\mu_{0}\delta_{0}^{-1}+\sum_{i\in \left[1,N\right]}\mu_{i}\delta_{i}^{-1}\right)\delta^{-1}$$

Based on this PoE framework, the multi‐omics ELBO extends the single‐modality ELBO (i.e., Equation (4)) by accounting for all omics data types (e.g., two modalities including RNA‐seq and DNA methylation data in this work). For a set of multi‐omics inputs the joint ELBO is expressed as:

(8) $$\text{ELBO}=\sum_{i=1}^{N}E_{q_{\phi }\left(z|x\right)}\left[\log p\left(x_{i}|z;\theta_{i}\right)\right]-\beta D_{\mathrm{KL}}\left(q_{\phi }\left(z|x\right)|p\left(z\right)\right)$$

Here, the first term, $\sum_{i=1}^{N}E_{q_{\phi }\left(z|x\right)}\left[\log p\left(x_{i}|z;\theta_{i}\right)\right]$ is the reconstruction loss across all modalities, measuring how well the model reconstructs each omics input $x_{i}$ from the joint latent variable z, with $\theta_{i}$ as the decoder parameters for modality i. The second term, $D_{\mathrm{KL}}\left(q_{\phi }\left(z|x\right)|p\left(z\right)\right)$ is the KL divergence between the joint variational posterior $q\left(z|x\right)$ and the prior $p\left(z\right)$, encouraging the joint latent space to stay close to the prior distribution. Utilizing a Product of Experts (PoE) framework, AOE‐Net constructs a joint latent representation, facilitating robust management of missing modalities during both pre‐training and fine‐tuning stages. In cases where only a single modality $x_{i}$ is available, the unobserved experts are effectively integrated out (or treated as uninformative), allowing the joint distribution to collapse into the single‐modal marginal distribution:

(9) $$q\left(z|X_{1:N}\right)\approx q\left(z|x_{i}\right)$$

By optimizing this multi‐omics ELBO, AOE‐Net learns a shared latent representation that captures shared patterns across diverse omics data.

### 4.2 Age‐Order‐Enhanced Contrastive Learning

To capture the aging‐related patterns across diverse omics data in generative modeling, the chronological age can be used to guide the distribution structure of latent representations. Considering that chronological age is provided as discrete 10‐year age bins (e.g., 3, 4, 5) in datasets like GTEx, an order‐enhanced contrastive learning is introduced to exploit this data structure reflecting ordinal age ranges. In this work, chronological age is discretized into K buckets with 10 years bins, and each sample's latent embedding $z_{i}\in R^{d}$ is paired with an age bucket label. The goal is to cluster representations or embeddings within age buckets while preserving ordinal relationships across age buckets. The corresponding loss function includes two components:

1. Loss of proxy‐based contrastive. A set of learnable proxies is introduced, where $p_{k}\in R^{d}$ (*k* represent one age bucket), represent each bucket centers. For embedding $z_{i}$ with label $a_{i}$, $p_{a_{i}}$ is the positive proxy, and others are negatives. The embedding similarity is computed as:

(10) $$s\left(z_{i},p_{k}\right)=\exp\left(\frac{\cos\left(z_{i},p_{k}\right)}{\tau }\right),\tau \;\text{is the hyperparameter},\text{and}\;\mathrm{set}\;\text{to}\;0.9\;\text{in default}$$

Negative samples are weighted dynamically by age difference:

(11) $$w_{i,k}=\frac{1}{1+\exp\left(-\frac{\left|a_{i}-k\right|}{\max_{k}\left|a_{i}-k\right|}\right)},\;w_{i,a_{i}}=0$$

The loss of proxy‐based contrastive is defined as follows:

(12) $$L_{\text{contrast}}=-\frac{1}{N}\sum_{i=1}^{N}\log\left(\frac{s\left(z_{i},p_{a_{i}}\right)}{s\left(z_{i},p_{a_{i}}\right)+\sum_{k\neq a_{i}}w_{i,k}s\left(z_{i},p_{k}\right)}\right)$$

2 Loss of order‐aware direction. To guarantee the expected ordinal consistency, direction vectors between embeddings $z_{i},z_{j}$ and proxies $p_{a_{i}},p_{a_{j}}$ are aligned:

(13) $$d_{i,j}=\text{normalize}\left(z_{i}-z_{j}\right),\;d_{i,j}^{\prime }=\text{normalize}\left(p_{a_{i}}-p_{a_{j}}\right)$$

The embedding similarity is computed as (τ is also 0.9 in default):

(14) $$s_{\mathrm{dir}}\left(i,j\right)=\exp\left(\frac{d_{i,j}\cdot d_{i,j}^{\prime }}{\tau }\right)$$

A mask $M_{i,j}=1$ if $a_{i}>a_{j}$; otherwise, 0; which guides the loss of order‐aware direction:

(15) $$L_{\text{direction}}=-\frac{1}{N}\sum_{i=1}^{N}\log\left(\frac{\sum_{j}M_{i,j}s_{\mathrm{dir}}\left(i,j\right)}{\sum_{j}s_{\mathrm{dir}}\left(i,j\right)}\right)$$

Finally, the combined loss for our age‐order‐enhanced contrastive learning is:

(16) $$L_{\text{order}}=L_{\text{contrast}}+\lambda L_{\text{direction}},\;\lambda =0.5$$

Proxies are initialized via Xavier uniform distribution and optimized during training. Ages are discretized into K=10 buckets with labels starting at 0 (e.g., stages 0–9).

### 4.3 Pre‐Training of AOE‐Net

By combining the ELBO with age‐enhanced contrastive learning, the final objective during pre‐training is:

(17) $$L_{\text{pretrain}}=\sum_{i=1}^{N}E_{q_{\phi }\left(z|x\right)}\left[\log p\left(x_{i}|z;\theta_{i}\right)\right]-\beta_{1}D_{\mathrm{KL}}\left(q_{\phi }\left(z|x\right)|p\left(z\right)\right)+\beta_{2}\;L_{\text{order}}$$

Here, $\beta_{1}$ and $\beta_{2}$ balanced the contributions of the multi‐omics data reconstruction, KL divergence regularization and age‐order‐enhanced learning.

### 4.4 Predicting Biological Age by Fine‐Tuning

AOE‐Net can be further fine‐tuned to estimate an individual's biological age using latent representations derived from either multi‐modal or single‐modal data in downstream studies. Pre‐trained to capture joint representations via PoE, AOE‐Net is adaptable for fine‐tuning on incomplete multi‐omics datasets depending on PoE, such as RNA‐seq or DNA methylation alone. The process for predicting biological age is outlined below. It produces outputs called logits, denoted as $l_{i}\in R^{B}\;\left(B=101\right)$ transformed via softmax to probabilities $p_{i}=\text{softmax}\left(l_{i}\right)$. The target distribution is a Gaussian centered at chronological age $a_{i}$:

(18) $$d_{i}\left(k\right)=\exp\left(-\frac{{\left(k-a_{i}\right)}^{2}}{2\sigma^{2}}\right)$$

The parameter σ controls the spread of the Gaussian distribution, determining how sharply or broadly the function decays as *k* deviates from $a_{i}$. In practice, the parameter σ can be adjusted during training to optimize model performance. Here, σ=3.0 is selected as a reasonable default, balancing sensitivity and smoothness. To ensure the target distributions are properly scaled, they can be normalized:

(19) $$d_{i}\left(k\right)=\frac{d_{i}\left(k\right)}{\sum_{k=1}^{B}d_{i}\left(k\right)+\epsilon },\;\epsilon =10^{-8}$$

The model's performance in fine‐tuning is evaluated by two measurements, whose loss functions consider cross‐entropy and regression.

1. Cross‐entropy loss. This measures how well the predicted probabilities match the target distribution:

(20) $$L_{\mathrm{CE}}=-\frac{1}{N}\sum_{i=1}^{N}\sum_{k=1}^{B}d_{i}\left(k\right)\log\left(p_{i}\left(k\right)\right)$$

2 Regression loss. This calculates the difference between the expected age ${\hat{a}}_{i}=\sum_{k=1}^{B}p_{i}\left(k\right)\cdot k$, and the actual age $a_{i}$:

(21) $$L_{\mathrm{reg}}=\frac{1}{N}\sum_{i=1}^{N}\left|{\hat{a}}_{i}-a_{i}\right|$$

Finally, the total loss should combine cross‐entropy and regression loss scores with a weighting factor β to balance their contributions:

(22) $$L=L_{\mathrm{CE}}+\beta L_{\mathrm{reg}},\beta \;\text{balanced the}\;\mathrm{two}\;\text{weights}$$

### 4.5 Personalized‐Context‐Aware Age Gap

The Personalized‐context‐aware Age Gap (PAAG) is proposed to quantify deviations in biological age relative to population norms. For each sample *i*, AOE‐Net outputs the biological age ${\hat{a}}_{i}$. Samples are grouped by chronological age a whose number is $N_{a}$, and the mean predicted biological age is:

(23) $$\mu_{\mathrm{bio}}\left(a\right)=\frac{1}{N_{a}}\sum_{i:a_{i}=a}\left({\hat{a}}_{i}\right)$$

The personalized‐context‐aware age gap for sample i is:

(24) $$g_{i}={\hat{a}}_{i}-\mu_{\mathrm{bio}}\left(a_{i}\right)$$

Positive $g_{i}$ indicates accelerated aging; negative values suggest slower aging. Furthermore, we divided the group into aging accelerated, normal and decelerated subgroups. The threshold ϵ was set as $\epsilon =\sigma_{g}$, where $\;\sigma_{g}$ is the standard deviation of the PAAG values $g_{i}$. Individuals were then classified into three groups: accelerated aging ($g_{i}>\epsilon$), normal aging ($\left|g_{i}\right|\leq \epsilon$), and decelerated aging $\left(g_{i}<-\epsilon \right)$.

### 4.6 Multi‐Omics Preprocessing

Following previous preprocess and filtering on RNA‐seq and DNA methylation (Wang et al. 2024), the gene expression data was represented as log2 (TPM + 1) and 11,816 features remain; while DNA methylation data excluded probes showing low methylation (beta‐value < 0.3) in more than 90% of samples and 6617 features remain for subsequent analysis.

### 4.7 SHAP Analysis for Feature Contribution

To ensure model interpretability and quantify the contribution of each molecular feature to the biological age prediction, we employed SHAP (SHapley Additive exPlanations). SHAP is a game‐theoretic approach that attributes an importance value to each feature for an individual prediction by calculating its marginal contribution across all possible feature coalitions. For each case study, we computed SHAP values for all input features (both RNA‐seq and DNA methylation) relative to the model's predicted biological age output using the SHAP Python package. To determine the overall importance of each feature, we calculated the mean absolute SHAP value across all samples. These global importance values were then used to rank features, allowing us to identify the top molecular contributors driving the model's predictions.

### 4.8 Functional Enrichment Analysis

To characterize the biological functions associated with influential genes, we performed Gene Ontology enrichment analysis using gprofiler2. The top 100 genes ranked by mean absolute SHAP value were used as the query set. Then we expanded this procedure by incorporating highly correlated or co‐expressed genes linked to the top 100 SHAP‐prioritized genes, thereby recovering biologically related features that might not appear among the top predictive variables because of redundancy or shared variance that may be discounted during model fitting. Enrichment analysis was performed in the GO Biological Process namespace, and terms with adjusted *p* < 0.05 were considered significant.

### 4.9 Baseline Aging Clocks

1. DeepMAge. It is a DNAm clock that utilizes a deep neural network (DNN) to predict chronological age from a wide array of DNAm sites (Galkin et al. 2021). Its non‐linear approach allows capturing complex patterns, resulting in high accuracy and strong associations with age‐related conditions. For a direct and fair comparison on our cohort, the DeepMAge model was retrained de novo on the TCGA pan‐cancer DNA methylation dataset, strictly following the methodology of the original publication.
2. BiT age clock. It is a transcriptomic clock (Meyer and Schumacher 2021), distinct from the other methylation‐based clocks. It employs a robust binary‐tree‐based ensemble learning approach to predict chronological age from gene expression (RNA‐seq) data. This method relies on a binarized signature of gene expression, making it resilient to noise. Consistent with evaluation for DeepMAge, the BiT age clock model was also retrained de novo using the corresponding TCGA pan‐cancer RNA‐seq expression data.
3. PhenoAge. This is a representative second‐generation DNAm clock (Levine et al. 2018) designed to capture biological aging rather than chronological age alone. It was trained on 513 CpGs to predict a composite score of biological age (Phenotypic Age) derived from clinical biomarkers, which is strongly associated with morbidity and mortality. We calculated PhenoAge using the established model within the methylclock R package.
4. HorvathAge. This is the foundational first‐generation pan‐tissue DNA methylation (DNAm) clock (Horvath 2013). It utilizes an elastic net regression model trained on 353 specific CpG sites to predict chronological age across diverse human tissues. In our study, HorvathAge was calculated using the original, pre‐trained model coefficients via the methylclock R package.
5. RNAAge. The transcriptomic‐based RNAAge score was calculated from the normalized RNA‐seq data using the RNAAgeCalc package (Ren and Kuan 2020).

### 4.10 Statistical and Metric Analysis

1. Hazard Ratio (HR). This is a measure used in survival analysis to compare the risk of an event (e.g., death or disease progression) between two groups, such as a treatment and control group for variables including age, PAAG in this study. It represents the ratio of the hazard rates—the instantaneous event rates at any given time—under the assumption of proportional hazards, as modeled by the Cox proportional hazards model. An HR greater than 1 indicates an increased risk in the treatment group relative to the control, while an HR less than 1 suggests a reduced risk, with HR equal to 1 implying no significant difference (Spruance et al. 2004).
2. The Concordance index (C‐index). This is a metric that evaluates the discriminative performance of a survival model by assessing the consistency between predicted risk scores and observed event times. It measures the proportion of all usable patient pairs—those with comparable event times—where the model's predicted risk order aligns with the actual outcome order (e.g., a patient with a shorter survival time is assigned a higher risk score). The C‐index ranges from 0 to 1, with 1 indicating perfect discrimination; 0.5 representing random guessing; and 0 signifying complete discordance. For all comparable pairs, a pair is deemed “concordant” if the patient with the shorter observed survival time has a higher predicted risk; and “discordant” otherwise. Then, the C‐index is computed as follows:

(25) $$C-\text{index}=\frac{\text{Number of concordant pairs}}{\text{Number of concordant pairs}+\text{Number of discordant pairs}}$$

Pairs with tied predictions or non‐informative outcomes may be excluded or assigned a fractional score (e.g., 0.5), depending on the implementation. The C‐index is commonly applied to assess the predictive accuracy of models like Cox regression or machine learning‐based survival predictors (Yan et al. 2013).

3 Multivariate logrank test (MLT). This test extends the traditional logrank test to compare survival distributions across multiple groups (e.g., different treatments or covariate levels) simultaneously. It tests the null hypothesis that the survival curves of all groups are identical against the alternative that at least one group differs, making it suitable for multi‐factor survival analysis. This test accounts for the overall difference in event rates across groups over time. The test statistic is calculated by aggregating observed and expected event counts across all groups at each event time. For k groups, let $O_{i}$ and $E_{i}$ represent the observed and expected number of events in group *i* respectively, with $E_{i}$ derived under the null hypothesis based on the risk set. The multivariate extension employs a vector $U=\left[O_{1}-E_{1},O_{2}-E_{2},\ldots ,O_{k}-E_{k}\right]$ and a covariance matrix V, computed from the risk sets and event distributions. The test statistic is:

(26) $$\chi^{2}=U^{T}V^{-1}U$$

This follows a chi‐square distribution with *k* − 1 degrees of freedom. A significant *p*‐value (e.g., *p* ≤ 0.05) indicates that survival distributions differ across groups. MLT is widely used in clinical trials and observational studies to evaluate multi‐group survival outcomes (Fay and Shaw 2010).

### 4.11 Implementation Details

The AOE‐Net model and PAAG computational pipeline are implemented using the PyTorch deep learning library in Python. In the pre‐training stage, the model is trained with the Adam optimizer, employing a learning rate of 1e‐4 and weight decay of 1e‐6, reduced by epoch. In the fine‐tuned stage, the multi‐omics encoder was updated with a learning rate of 1e‐6, and the MLP age estimator with a learning rate of 1e‐3. Model training was conducted over 50 epochs, with early stopping triggered after 10 steps of no improvement. All experiments were conducted on a workstation equipped with two Intel Xeon Silver 4210R CPUs and one NVIDIA GeForce RTX 4090 GPU.

## Author Contributions

Y.L. and T.Z. conceived the project. T.Z. and F.‐A.W. designed the framework and analysis. Y.Y., E.D., C.Y., H.W., J.J., J.C. collected and preprocessed the multi‐omics data. F.‐A.W. carried out the experiments. Y.Y., C.Y., H.W. made analysis and discussion. Y.L., D.T., Y.W., R.H. provided resources. F.‐A.W. and T.Z. wrote the manuscript with contributions from all authors. T.Z. supervised the entire project. All authors read and approved the final manuscript.

## Funding

This work was supported by the National Key R&D Program of China (2022YFF1202100 and 2023YFF1204700), the National Natural Science Foundation of China (92574105 and 12371485), the Major Project of Guangzhou National Laboratory (GZNL2025C01013), and the Natural Science Foundation of Guangdong Province of China (2025A1515011988).

## Conflicts of Interest

The authors declare no conflicts of interest.

## Supporting information


**Figure S1:** Diagram of traditional age gap and population‐aware age gap based on TCGA data. (a) The diagram of traditional age gap, the difference between predicted biological age and chronological age. (b) The diagram of personalized‐context‐aware age gap, the difference between the predicted biological age and the mean biological age of the same group.
**Figure S2:** Age‐informed latent trajectories captured by AOE‐Net. (a) UMAP projection of latent embeddings colored by age bucket labels, highlighting the distribution of samples across distinct age groups/populations. (b) UMAP projection of latent embeddings colored by pseudotime, illustrating the progression of samples along a pseudotemporal trajectory captured by AOE‐Net.
**Figure S3:** Proteomic augmentation modestly improves prognostic performance of AOE‐Net. Comparison of C‐index values for survival prediction using the bi‐modal model (RNA + methylation) and the tri‐modal model (RNA + methylation + protein [RPPA]) across young, middle, and old age subgroups.
**Figure S4:** Tri‐modal AOE‐Net‐derived PAAG stratifies survival across chronological age subgroups. Kaplan–Meier overall survival curves for decelerated, normal, and accelerated aging groups defined by PAAG from the tri‐modal (RNA + methylation + protein) AOE‐Net in young (< 40), middle (40–60), and old (> 60) pan‐cancer subgroups.
**Figure S5:** Senescence‐associated gene expression across PAAG‐defined aging groups in the pan‐cancer cohort. Violin plots showing expression of six senescence‐related genes across decelerated, normal, and accelerated aging groups defined by PAAG.
**Figure S6:** Senescence signature score across PAAG‐defined aging groups. Violin plot showing the senescence signature score across decelerated, normal, and accelerated aging groups in the pan‐cancer cohort.
**Figure S7:** Distribution of SHAP values and expression levels for genes, and SHAP values for DNA methylation CpG sites in TCGA pan‐cancer cohorts.
**Figure S8:** Association between PAAG and DunedinPACE in the pooled TCGA pan‐cancer cohort. Density scatter plot, which show the relationship between PAAG and DunedinPACE across pooled TCGA pan‐cancer samples. Each dot represents one sample, colored by point density.
**Figure S9:** Cancer‐specific heterogeneity in the association between PAAG and DunedinPACE. Forest plot showing cancer‐specific linear slopes for the association between PAAG and DunedinPACE across TCGA cancer types with sample size > 100.
**Figure S10:** Distribution of SHAP and methylation values for CPG sites, and SHAP values for DNA methylation CpG sites in osteoporosis individuals.

**Table S1:** Limma results for the gene expression changes between individuals with no disease (n=136) vs individuals with generalized SA (n=146) using transcriptomics data.

## Data Availability

The GTEx, TCGA, and PESA cohorts employed in this study are all publicly accessible. The RNA‐seq data of GTEx can be downloaded at https://www.gtexportal.org/home/. The pre‐processed multi‐omics of TCGA are available at https://xena.ucsc.edu/. The tumor immune response data can be collected at https://gdc.cancer.gov/about‐data/publications/panimmune. The RNA‐seq data of the TARGET (Genomics) cohort are publicly available at https://treehousegenomics.soe.ucsc.edu/public‐data/. The PESA cohorts can be found in the Gene Expression Omnibus (GEO) at https://www.ncbi.nlm.nih.gov/geo/ and can be accessed with accession numbers GSE220622 and GSE221615. The DNA methylation profiles of the osteoporosis cohort used in this study can be accessed in NODE (http://www.biosino.org/node) by entering the accession (OEP003842) into the text search box or through the URL: http://www.biosino.org/node/project/detail/OEP003842. The RPPA dataset used for tri‐modal validation is accessible through the TCPA data portal (https://tcpaportal.org). The supplementary data can be accessed at https://github.com/FengAoWang/AgingAOE‐Net/blob/main/dataset. The AOE‐Net model was implemented using Python and Pytorch deep learning framework, and all source code utilized in this study is publicly available on GitHub (https://github.com/FengAoWang/AgingAOE‐Net).
