# Supplementary material for: Personalized‐Context‐Aware Age Gap: A New Multi‐Omics Measurement Based on Age‐Enhanced Model AOE‐Net for Aging Acceleration and Chronic Disease Risk Prediction
Source: Aging Cell. 2026 May 28;25(6):e70552. doi: 10.1111/acel.70552 (PMC13240351; doi:10.1111/acel.70552)
Supplement: Supplementary file 1 — Figure S1: Diagram of traditional age gap and population‐aware age gap based on TCGA data. (a) The diagram of traditional age gap, the difference between predicted biological age and chronological age. (b) The diagram of personalized‐context‐aware age gap, the difference between the predicted biological age and the mean biological age of the same group. Figure S2: Age‐informed latent trajectories captured by AOE‐Net. (a) UMAP projection of latent embeddings colored by age bucket labels, highlighting the distribution of samples across distinct age groups/populations. (b) UMAP projection of latent embeddings colored by pseudotime, illustrating the progression of samples along a pseudotemporal trajectory captured by AOE‐Net. Figure S3: Proteomic augmentation modestly improves prognostic performance of AOE‐Net. Comparison of C‐index values for survival prediction using the bi‐modal model (RNA + methylation) and the tri‐modal model (RNA + methylation + protein [RPPA]) across young, middle, and old age subgroups. Figure S4: Tri‐modal AOE‐Net‐derived PAAG stratifies survival across chronological age subgroups. Kaplan–Meier overall survival curves for decelerated, normal, and accelerated aging groups defined by PAAG from the tri‐modal (RNA + methylation + protein) AOE‐Net in young (< 40), middle (40–60), and old (> 60) pan‐cancer subgroups. Figure S5: Senescence‐associated gene expression across PAAG‐defined aging groups in the pan‐cancer cohort. Violin plots showing expression of six senescence‐related genes across decelerated, normal, and accelerated aging groups defined by PAAG. Figure S6: Senescence signature score across PAAG‐defined aging groups. Violin plot showing the senescence signature score across decelerated, normal, and accelerated aging groups in the pan‐cancer cohort. Figure S7: Distribution of SHAP values and expression levels for genes, and SHAP values for DNA methylation CpG sites in TCGA pan‐cancer cohorts. Figure S8: Association between PAAG and Dunedin [file ACEL-25-e70552-s002.docx]

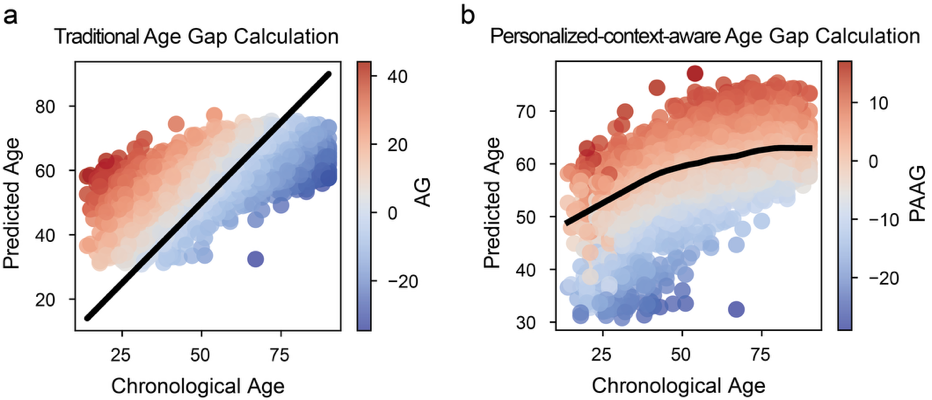


**Figure. S1** Diagram of traditional age gap and population-aware age gap based on TCGA data. **a.** The diagram of traditional age gap, the difference between predicted biological age and chronological age. **b.** The diagram of personalized-context-aware age gap, the difference between the predicted biological age and the mean biological age of the same group.


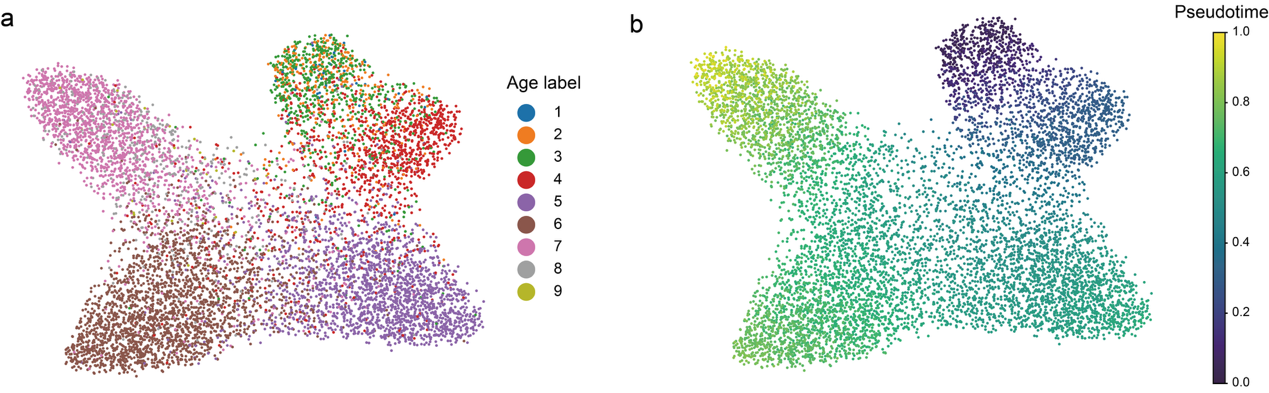


**Figure. S2** Age-informed latent trajectories captured by AOE-Net. **a.** UMAP projection of latent embeddings colored by age bucket labels, highlighting the distribution of samples across distinct age groups/populations. **b.** UMAP projection of latent embeddings colored by pseudotime, illustrating the progression of samples along a pseudotemporal trajectory captured by AOE-Net.


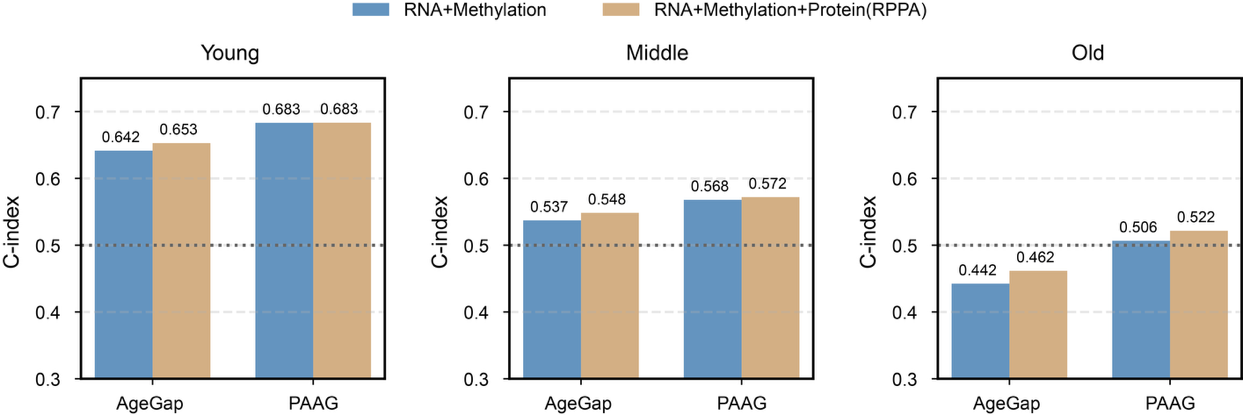


**Figure. S3** Proteomic augmentation modestly improves prognostic performance of AOE-Net. Comparison of C-index values for survival prediction using the bi-modal model (RNA + methylation) and the tri-modal model (RNA + methylation + protein [RPPA]) across young, middle, and old age subgroups.


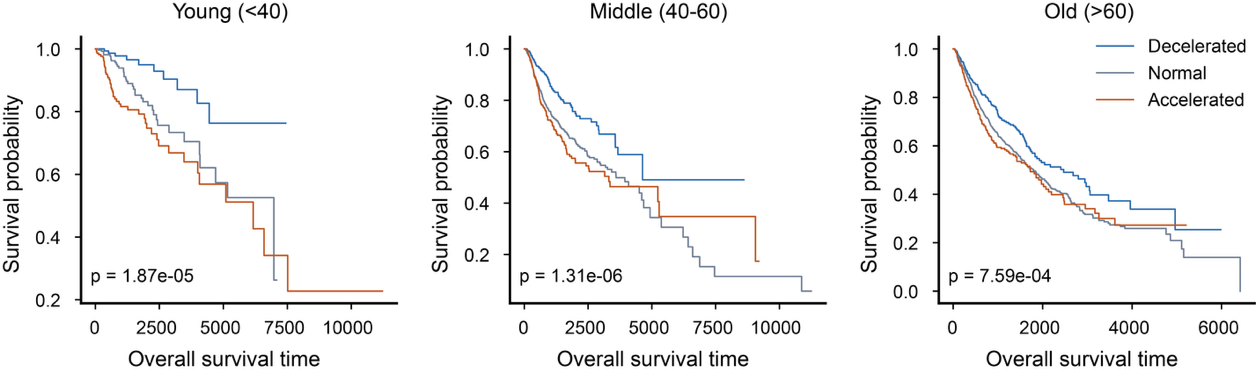


**Figure. S4** Tri-modal AOE-Net-derived PAAG stratifies survival across chronological age subgroups. Kaplan-Meier overall survival curves for decelerated, normal, and accelerated aging groups defined by PAAG from the tri-modal (RNA + methylation + protein) AOE-Net in young (<40), middle (40-60), and old (>60) pan-cancer subgroups.

**
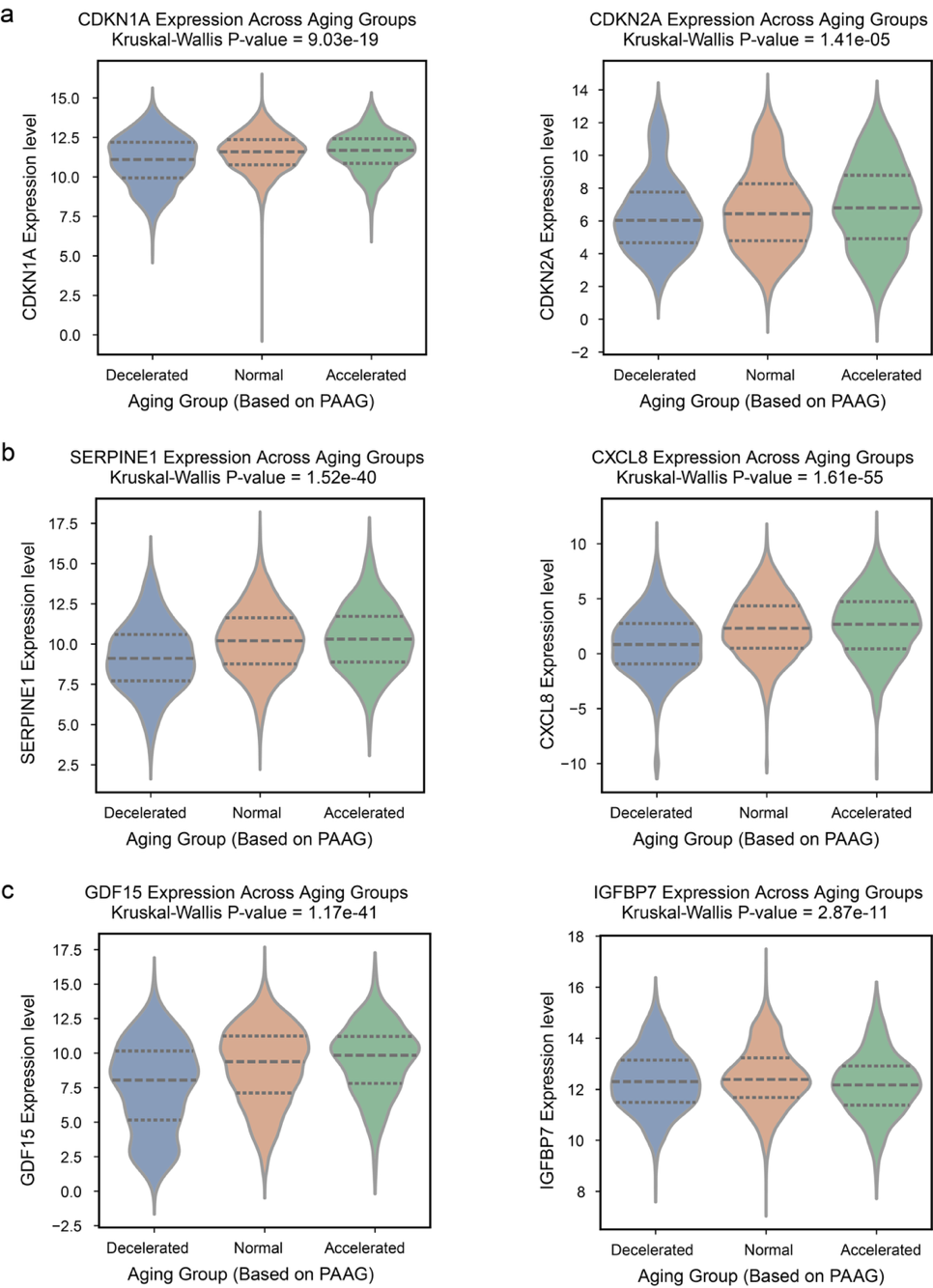
**

**Figure. S5** Senescence-associated gene expression across PAAG-defined aging groups in the pan-cancer cohort. Violin plots showing expression of six senescence-related genes across decelerated, normal, and accelerated aging groups defined by PAAG.


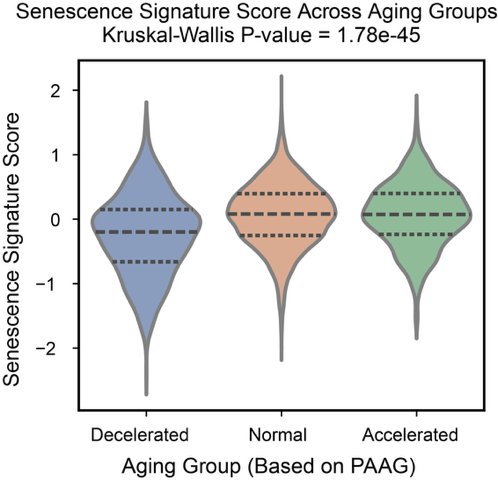


**Figure. S6** Senescence signature score across PAAG-defined aging groups. Violin plot showing the senescence signature score across decelerated, normal, and accelerated aging groups in the pan-cancer cohort.


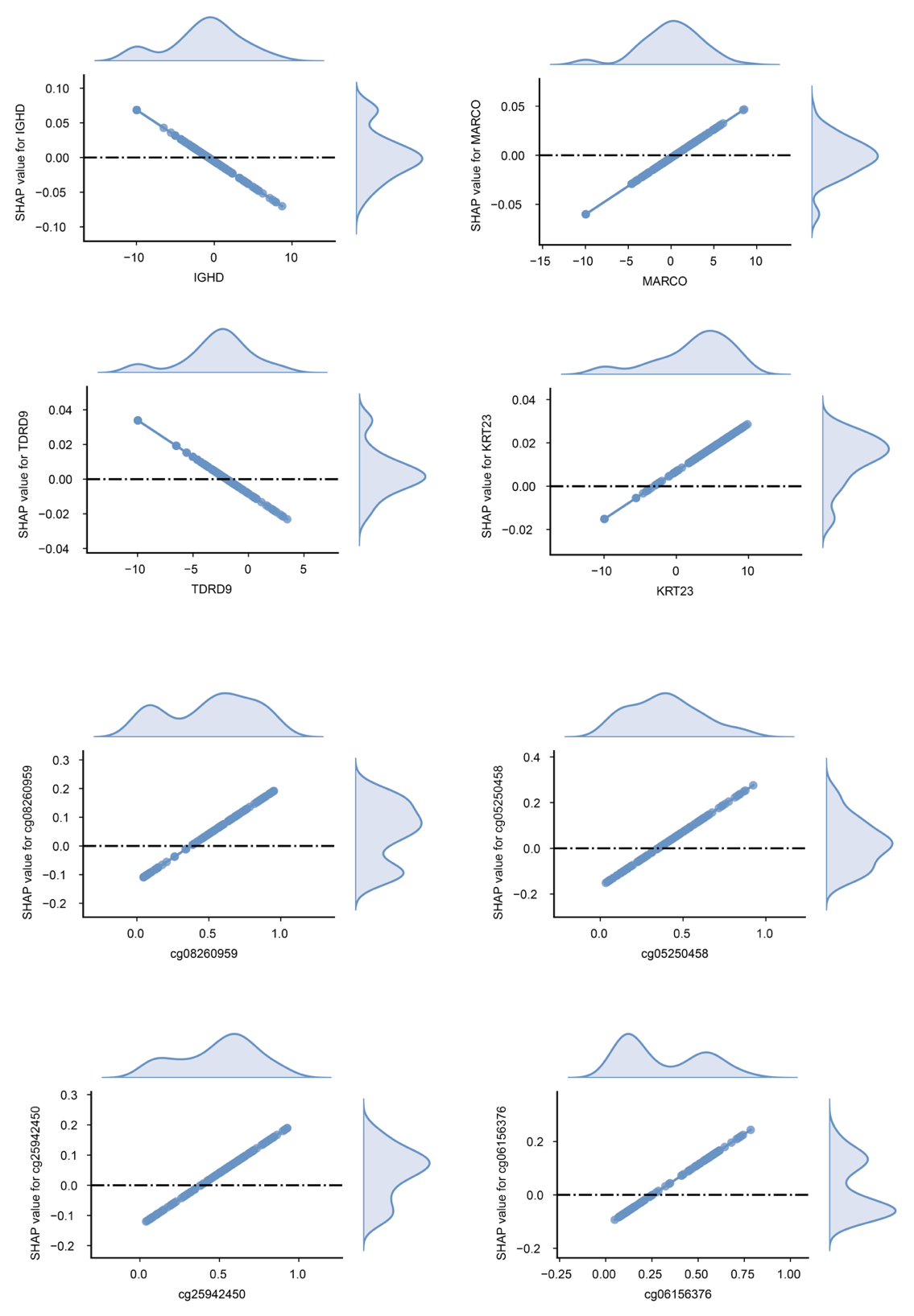


**Figure. S7** Distribution of SHAP values and expression levels for genes, and SHAP values for DNA methylation CpG sites in TCGA pan-cancer cohorts.


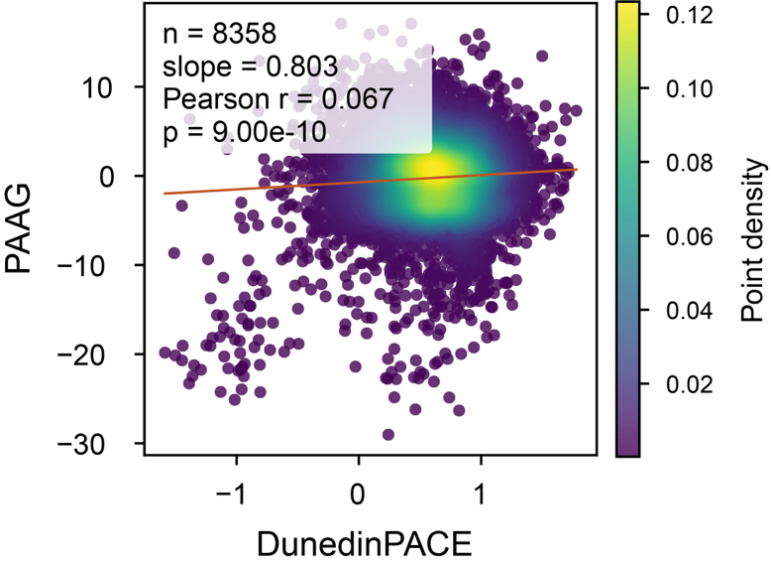


**Figure. S8** Association between PAAG and DunedinPACE in the pooled TCGA pan-cancer cohort. Density scatter plot, which show the relationship between PAAG and DunedinPACE across pooled TCGA pan-cancer samples. Each dot represents one sample, colored by point density.


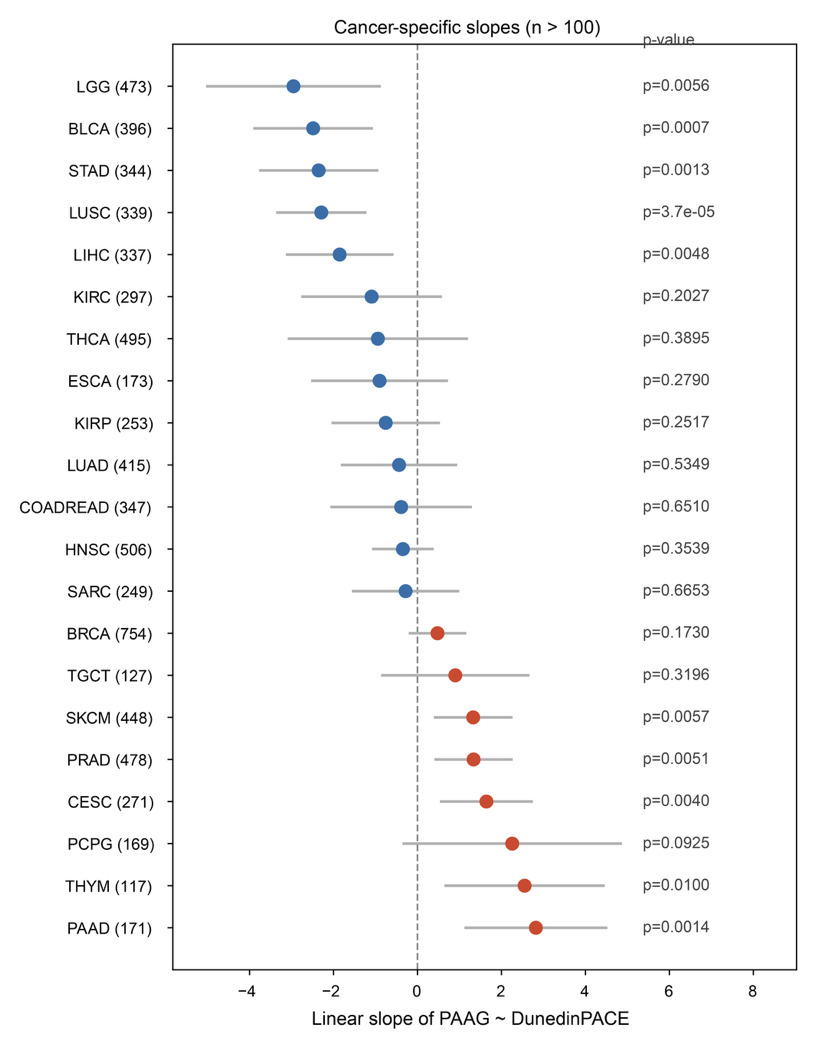


**Figure. S9** Cancer-specific heterogeneity in the association between PAAG and DunedinPACE. Forest plot showing cancer-specific linear slopes for the association between PAAG and DunedinPACE across TCGA cancer types with sample size >100.


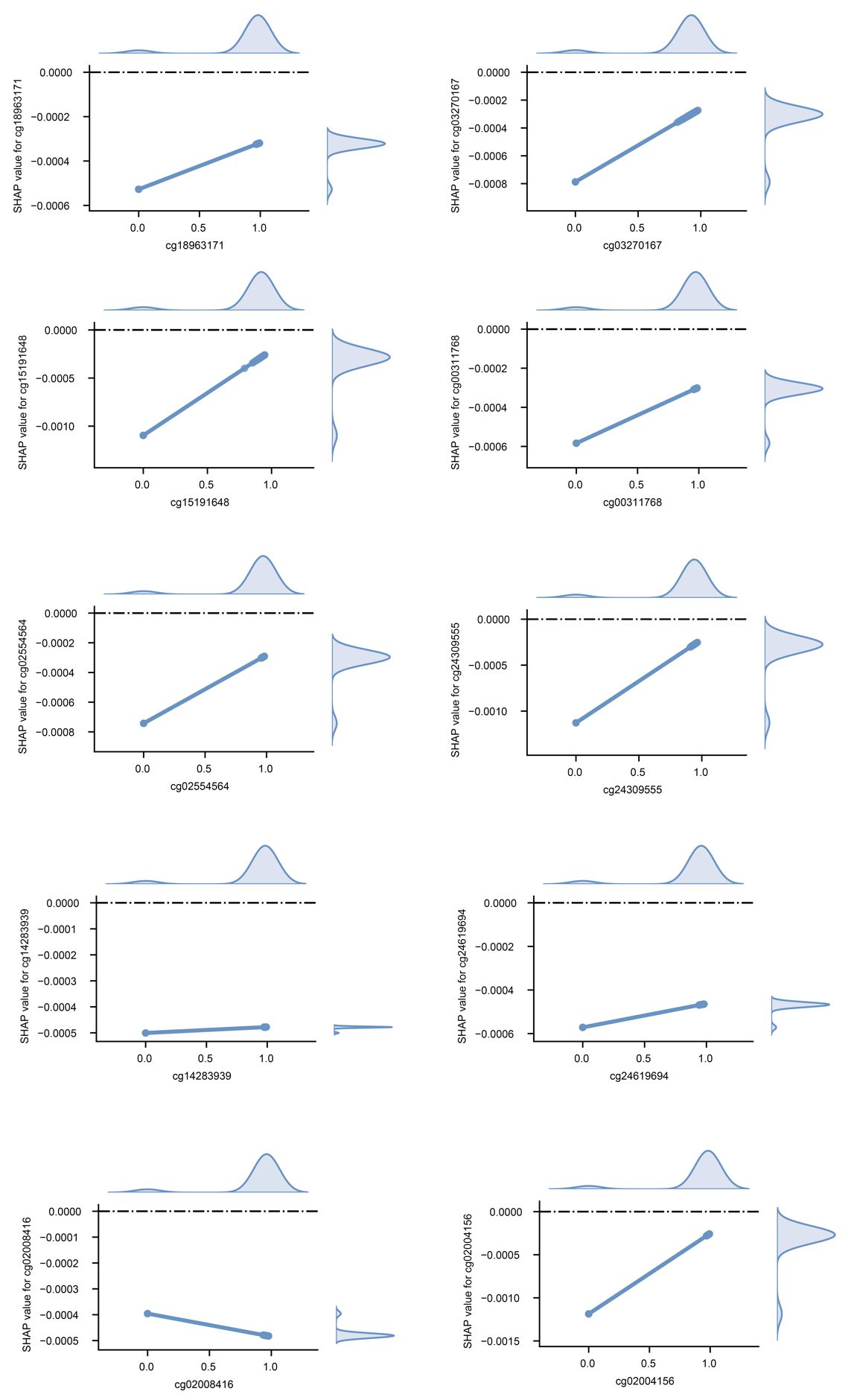


**Figure. S10** Distribution of SHAP and methylation values for CPG sites, and SHAP values for DNA methylation CpG sites in osteoporosis individuals.
